# Genome-Wide Identification of Discriminative Genetic Variations in Beef and Dairy Cattle via an Information-Theoretic Approach

**DOI:** 10.3390/genes11060678

**Published:** 2020-06-22

**Authors:** Soo-Jin Kim, Jung-Woo Ha, Heebal Kim

**Affiliations:** 1 Department of Agricultural Biotechnology and Research Institute of Agriculture and Life Sciences, Seoul National University, Seoul 08826, Korea; kim.soojin@outlook.com; 2 Clova AI Research, NAVER Corp., Seongnam 13561, Korea; jungwoo.ha@navercorp.com; 3 Interdisciplinary Program in Bioinformatics, Seoul National University, Seoul 08826, Korea; 4 C&K Genomics, Seoul 05836, Korea

**Keywords:** cattle genome-wide analysis, conditional mutual information, Angus and Jersey cattle, genetic variations, single nucleotide polymorphisms

## Abstract

Analyzing the associations between genotypic changes and phenotypic traits on a genome-wide scale can contribute to understanding the functional roles of distinct genetic variations during breed development. We performed a whole-genome analysis of Angus and Jersey cattle breeds using conditional mutual information, which is an information-theoretic method estimating the conditional independency among multiple factor variables. The proposed conditional mutual information-based approach allows breed-discriminative genetic variations to be explicitly identified from tens of millions of SNP (single nucleotide polymorphism) positions on a genome-wide scale while minimizing the usage of prior knowledge. Using this data-driven approach, we identified biologically relevant functional genes, including breed-specific variants for cattle traits such as beef and dairy production. The identified lipid-related genes were shown to be significantly associated with lipid and triglyceride metabolism, fat cell differentiation, and muscle development. In addition, we confirmed that milk-related genes are involved in mammary gland development, lactation, and mastitis-associated processes. Our results provide the distinct properties of Angus and Jersey cattle at a genome-wide level. Moreover, this study offers important insights into discovering unrevealed genetic variants for breed-specific traits and the identification of genetic signatures of diverse cattle breeds with respect to target breed-specific properties.

## 1. Introduction

Manipulating domesticated animals by inbreeding and artificial selection has led to the development of a multitude of individual cattle breeds. As a result, many cattle breeds have become highly specialized for meat or milk production subsequent to strong genetic selection for these traits. In this context, investigating the associations between genetic variations and phenotypes has significant potential for understanding the heritability of complex traits in cattle. Moreover, such a study will identify distinct genetic factors that are likely to relate to breed-specific characteristics. 

Meat and milk yield are important economic factors for cattle production, and Angus and Jersey cattle are representative breeds for beef and dairy traits, respectively. The Angus breed has been intensively selected over the last few decades to reduce several recessive genetic disorders [1]. Jersey cattle were originally bred on the British Channel Island of Jersey, and a number of Jersey breeds have become highly specialized for milk production factors, such as high butterfat content [2]. Thus, analyzing the genetic profiles of Angus and Jersey breeds can aid in investigating important economic traits such as meat and milk production. Recently, the accumulation of massive genotype information on numerous cattle breeds has facilitated detailed studies of cattle genetic variants for the design and development of livestock. A large-scale analysis of the bovine genome may also have an impact on cattle farming by providing new insights for cattle breeding and production programs. 

Several studies have been performed to obtain evidence of selection on a genome-wide level in cattle [3,4,5,6,7,8], and various statistical methods have been successfully proposed to detect selection signatures from genetic polymorphism data. The allele frequency spectrum and haplotype segregation are key concepts for inferring the signatures of selection in populations. The fixation index (F_ST_) [9] and the cross-population composite likelihood ratio (XP-CLR) [10] are based on variations in the allele frequencies in populations for detecting genomic regions under selection. Linkage information is also employed to identify selection signatures in populations by investigating long-range haplotypes. Long-range haplotype methods use the integrated haplotype score (iHS) [11] and the across-population extended haplotype homozygosity (XP-EHH) [12] to identify alleles segregating in a population based on haplotype length. These methods have relied on variation patterns (e.g., allele frequencies or long haplotypes) as constraints for efficient measurements; however, they may cause SNPs to dominate which are indirectly or implicitly related to these constraints, because they are very sensitive to SNP ascertainment bias [10,13].

In this study, we performed a comparative genome analysis of two cattle breeds, Angus and Jersey, using an information-theoretic approach. The proposed mutual information extractor based on conditional mutual information (CMI) is a principled data-driven method and minimizes the use of prior knowledge to identify discriminative SNPs determining breed-specific traits, such as meat and milk yield. Thus, our analysis focuses on systematically detecting the distinct SNPs that discriminate cattle breeds on a genome-wide scale. Together with detecting new genetic patterns from cattle genome, we found putative genes showing genetic signatures that may have contributed to the development of Angus- and Jersey-specific phenotypes, such as beef and dairy production.

## 2. Materials and Methods

### 2.1. Sequencing, Quality Control, and Variant Calling

We collected whole-blood samples (10 mL) from 10 Angus cattle and 10 Jersey cattle. The Angus and Jersey samples originated from Chamtowoo (Seoul, Korea), the Seoul Milk Cooperative (Yangpyeong, Gyeonggi, Korea), and the Korea Federation of Livestock Cooperatives (Dangjin, Chungnam, Korea). The blood samples were obtained from jugular veins. The DNA was extracted using the Wizard Genomic DNA Purification Kit (Promega, Seoul, Korea). The collection of blood samples was performed in accordance with the guidelines given by the relevant agricultural institutions. All methods involving animal works were approved by the Institutional Animal Care and Use Committee of the National Institute of Animal Science (NIAS) in Korea under approval numbers NIAS-2014-093. 

We produced pair-end reads using an Illumina HiSeq 2000 and isolated DNA from whole blood using a G-DEXTMIIb Genomic DNA Extraction Kit (iNtRoN Biotechnology, Seongnam, Korea) following the instructions of the manufacturer. We used the Covaris System to generate 3 µg of genomic DNA for generating the ~300 bp inserts. The fragments of the shared DNA were end-repaired, A tailed, adaptor-ligated, and amplified using the TruSeq DNA Sample Prep. Kit (Illumina, San Diego, CA, USA). Paired-end sequencing was performed on the Illumina HiSeq 2000 platform using the TruSeq SBS Kit v3-HS (Illumina, San Diego, CA, USA) with NICEM (National Instrumentation Center for Environmental Management, Seoul, Korea). Raw Sequence data are available from the National Centre for Biotechnology Information (NCBI) with the Bioproject accession numbers PRJNA318087 (Angus) and PRJNA318089 (Jersey).

A per-base sequence was quality checked with fastQC, which calculates various quality metrics for raw reads. Next, the pair-end sequence reads were aligned to a bovine reference genome (UMD 3.1) using Bowtie2 [14]. We used default parameters (except the “-no-mixed” option) to inhibit unpaired alignments for paired reads.

We used open-source software packages for downstream processing and variant calling. Potential PCR duplicates were filtered using the “REMOVE_DUPLICATEDS = true” option in “MarkDuplicates” of Picard. We then used SAMtools [15] to generate index files for the reference and bam files. GATK [16] was used to correct misalignments due to the presence of indels by performing a local realignment of reads with “RealignerTargetCreator” and “IndelRealigner” modules. The “UnifiedGenotyper” and “SelectVariants” modules of GATK were used for calling candidate SNPs. Next, we used the “VariantFiltration” of GATK to filter variants and avoid possible false positives with the following options: SNPs with QUAL (Phred-scaled quality score) < 30 were filtered; SNPs with MQ0 (the number of reads with a mapping quality of zero across all samples) > 4 and QD (variants confidence/quality by depth; low scores are indicative of false positives and artifacts) < 5 were filtered, and SNPs with FS (Phred-scaled *p*-value using Fisher’s exact test) > 200 were filtered. We used BEAGLE [17] to impute missing genotypes and infer haplotype phases for the whole set of cattle populations simultaneously.

### 2.2. Conditional Mutual Information 

Information theory has provided a theoretical basis in many data analysis and machine learning tasks since it was proposed for communication and compression perspectives [18,19]. In particular, mutual information (MI) has been a metric widely applied for extracting significant variables from high dimensional data, including gene expression and sequencing data [20,21]. Since it is defined with the entropy of random variables similar to the other information-theoretical methods, such as Kullback–Leibler divergence (KL-divergence), MI formulates the conditional independency between two random variables. 

It is straightforward to apply MI to select significant genetic factors associated with cattle breed, and MI can be used to calculate the associations between the SNP positions and breeds. Assuming that genetic factors (e.g., SNP) and class variables (e.g., cattle breed) are random variables, MI can formulate the dependency of each genetic factor on cattle trait. In formal, let *x* and *y* denote an SNP position and breed variables. Given an SNP position and breed variables, MI between the SNP position and cattle breed, *I*(*x*;*y*), is defined with the entropy of *x* and the conditional entropy of *x* and *y* as follows:

I(x;y)=H(x)−H(x|y),

$$I(x;y)=\sum_{x\in X}\sum_{y\in Y}p(x,y)\log\frac{p(x,y)}{p(x)p(y)},\;s.t.\;H(x)=-\sum_{x\in X}p(x)\log p(x),\;\mathrm{and}\;H(x|y)=H(x,y)-H(y),$$

where *X* and *Y* denote the SNP position and the breed variable sets, respectively. When *I*(*x*;*y*) is equal to 0, the SNP corresponding to *x* is independent of breed, and it means that the SNP does not influence breed in the aspect of a pairwise relationship. 

Since MI is a metric for representing the independency between two variables, it is not trivial to characterize the effects of multiple factors on determining cattle breed. To solve this issue, in this study, we employed conditional mutual information (CMI) among two SNP variables and a breed variable as the criterion for extracting important SNP positions [22,23]:

$$CI(y;x_{1}|x_{2})=CI(y;x_{1},x_{2})-CI(y;x_{2}),$$

where *x*_1_ and *x*_2_ are two SNP variables. 

We are interested in *CI*(*y*; *x*_1_, *x*_2_) and call it the mutual information extractor in the rest of this paper. Mutual information extractors can be defined from the chain rule for mutual information:

$$CI(y;x_{1},x_{2})=CI(y;x_{1}|x_{2})+CI(y;x_{2}),\;s.t.\;CI(y;x_{1}|x_{2})=\sum_{s_{1}\in x_{1}}\sum_{s_{2}\in x_{2}}\sum_{y\in Y}p_{Y,x_{1},x_{2}}(y,s_{1},s_{2})\log\frac{p_{x_{2}}(s_{2})p_{Y,x_{1},x_{2}}(y,s_{1},s_{2})}{p_{Y,x_{2}}(c,s_{2})p_{x_{1},x_{2}}(s_{1},s_{2})},$$

where *s*_n_ is the allele value of the *n*-th SNP *x*_n_. 

A mutual information extractor quantifies the associations between SNPs at two loci and breeds. Since *CI*(*y*; *x*_1_, *x*_2_) is nonnegative, the same for mutual information, the mutual information extractor is equal to 0 when all three variables are conditionally independent. This property is suitable for detecting discriminative two-locus haplotypes influencing cattle breed. Thus, our method is effective in detecting the discriminative SNPs showing a high dependence between the haplotypes of two adjacent loci and breed.

### 2.3. XP-CLR and XP-EHH Tests

We performed cross-population composite likelihood ratio (XP-CLR) and cross-population extended haplotype homozygosity (XP-EHH) tests for detecting the selective signatures in Angus and Jersey cattle. These two statistics are representative methods that use different criteria to detect the genomic regions under selection in populations. The XP-CLR statistic is able to detect regions of rapid changes in allele frequency at a locus with random drift [10]. In contrast, the XP-EHH statistic is designed to identify nearly fixed selective signatures by comparing the haplotypes of two populations by measuring linkage disequilibrium [12].

The XP-CLR test is based on the detection of multi-locus allele frequency differentiation across populations, which is not as affected by ascertainment bias [10]. We used the following parameters: non-overlapping sliding windows of 50 kb, a maximum number of SNPs within each window of 400, and the correlation level of the SNPs’ contribution to the XP-CLR results down-weighted to 0.95. The regions with XP-CLR values in the top 1% of the empirical distributions in the Angus and Jersey samples were designated as candidate sweeps. 

In addition, the XP-EHH is designed to find alleles with an increase in frequency to the point of fixation or near-fixation in one of the populations by comparing haplotypes from two populations [12]. It means that it detects SNPs which are under selection in one population but not in others. So, the extreme XP-EHH scores potentially describe the selection of a particular population. We computed the EHH and the log-ratio values of the iHH (integrated EHH) for the pairwise test of the Angus and Jersey populations. The log ratios were normalized to have a mean of 0 and a variance of 1. In addition, XP-EHH scores are directional. A positive score indicates that selection is likely to have happened in population A, while a negative score means the selection probably occurs in population B. In this study, an XP-EHH value indicating a positive score suggests selection in Angus cattle, whereas a negative score signifies selection in Jersey cattle. We selected the regions with XP-EHH scores in the top and bottom 1% of the empirical distributions (empirical *p*-value < 1.0 × 10^−3^), and the selected genomic regions were annotated to the closest genes. 

## 3. Results

**Figure 1:**
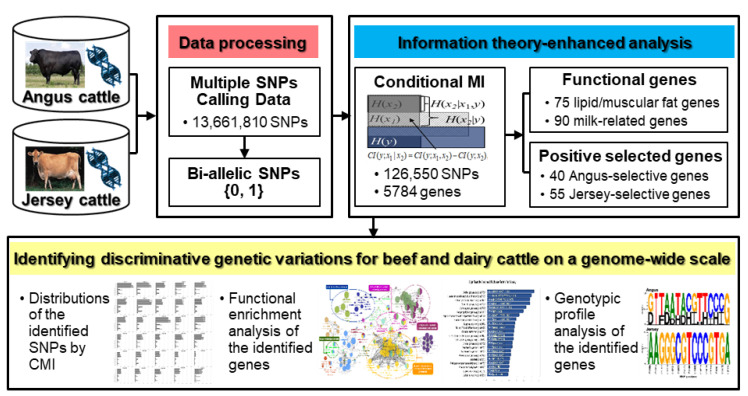
Schematic overview of information theory-enhanced analysis on cattle genome for identifying discriminative genetic variations between Angus and Jersey.

We performed a comparative genome-wide analysis for identifying discriminative genetic variations between Angus and Jersey cattle using enhanced methods based on the information-theoretic approach (Figure 1).

### 3.1. SNP Detection

The genomes of the Angus and Jersey cattle were sequenced to approximately 15.79× coverage on average, with a total of 840,132,997,679 bp in 8,401,720,919 reads. The pair-end sequence reads were aligned with an average alignment rate of 97.54%, and the reads covered 98.82% of the genome across all of the samples on average (Table S1). A total of ~13 million SNPs were obtained after filtering the potential PCR duplicates and correcting misalignments (Table S2). 

### 3.2. Population Structures

Principal component analysis (PCA), a linear dimensionality reduction method, is broadly used to extract the fundamental structure of a dataset via the projection of individuals into a subspace spanned by the largest principal components [24,25]. In genetics, given that there are a large number of SNPs for many individuals, PCA can be applied to infer the patterns of population structure. To detect the genetic structure of populations, we conducted a PCA on SNP genotype data extracted from Angus and Jersey breed samples via genome-wide complex trait analysis (GCTA) [26] as implemented in EIGENSTRAT [24]. The analysis disregards breed membership but, nevertheless, displays clear breed structures as samples from the same breed cluster together. The Angus samples are separated from the Jersey samples in the projection subspace (the largest PC was 19.06% of the total variation), as shown in Figure S1. This separation indicates that these two breeds show no evidence of admixture with each other.

### 3.3. Extraction of Discriminative SNPs Based on the Information-Theoretic Method 

We used a mutual information extractor for the Angus and Jersey breeds to identify the candidate SNPs with discriminative potential. We extracted 126,550 SNPs annotated to as 5874 genes (Figure S2 and Table S2). Table S3 shows the detailed distribution of the number of the extracted SNPs on each chromosome. The extracted SNPs have high CMI values (θ = 0.693) at a significant level (*p*-value equal to 2.98 × 10^−5^). To overcome any bias caused by the small number of samples, we set a strict *p*-value for estimating statistical significance (*p*-value less than 1.0 × 10^−3^) compared with those used in other studies [27].

**Figure 2:**
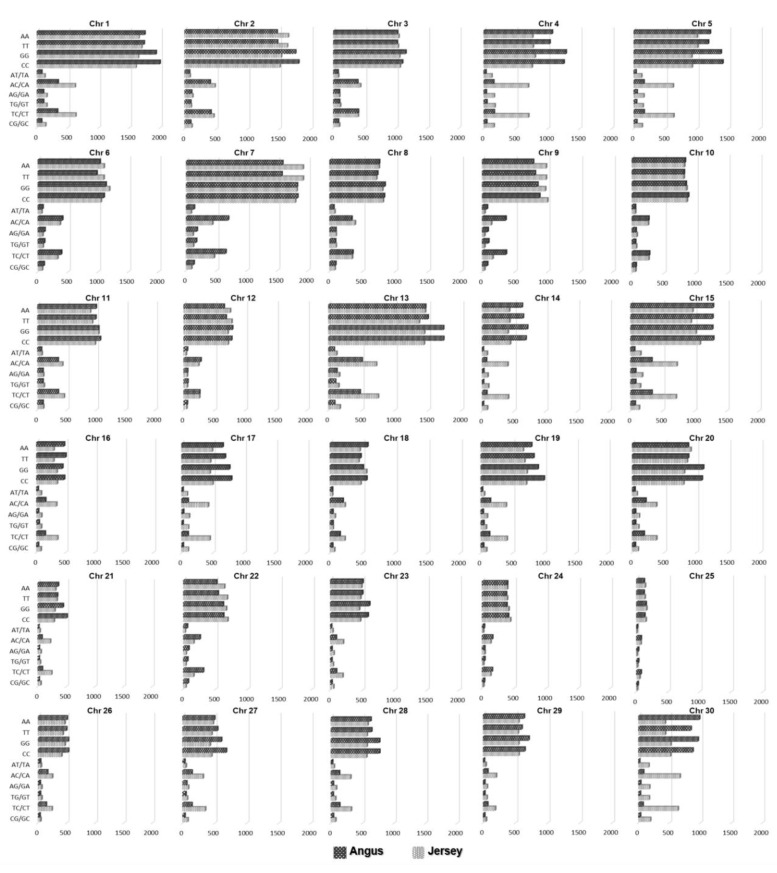
Distributions of the SNPs identified by conditional mutual information (CMI) distinguishing between Angus and Jersey on each chromosome. Patterned black and grey bars indicate the numbers of SNPs identified by CMI in Angus and Jersey according to the genotypes of SNPs on each chromosome. For all graphs, the *x*-axis is the number of SNPs, and the *y*-axis represents the genotypes of SNPs (AA, TT, GG, CC, AT/TA, AG/GA, AC/CA, TG/GT, TC/CT, and GC/CG).

Figure 2 shows the distributions of the SNPs identified by CMI distinguishing between two breeds on each chromosome, excluding the mitochondrial genome. It presents the distributions of the identified SNPs on each chromosome for AA, TT, GG, CC, AT/TA, AG/GA, AC/CA, TG/GT, TC/CT, and GC/CG genotypes, respectively, in Angus and Jersey. Figure S3 is the graph of the ratio of the SNPs identified by CMI with a significant *p*-value to total the SNPs across all chromosomes. The result shows that the distribution of SNP extracted by mutual information extractor was different in Angus versus Jersey cattle. These distributions of the SNPs on each chromosome can provide the information on genomic locations that are likely to have received selection pressure and possess the ability to distinguish Angus and Jersey breeds. Hence, the regions containing the extracted SNPs can offer specific candidate areas for a fine-grained mapping of the genes that are important for discriminating between the two breeds.

**Figure 3:**
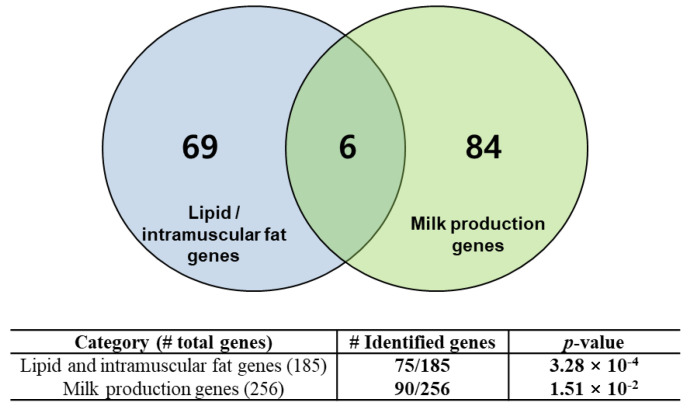
Functional genes identified based on conditional mutual information. The identified genes were enriched in lipid/intramuscular fat genes or mammary gland/milk production-related genes with significant *p*-value levels.

Many of the genes mapped in the regions of the extracted SNPs were highly associated with functional genes for beef and dairy traits. To evaluate this finding, we collected a list of 185 lipid and intramuscular fat-related genes and 256 mammary gland/milk-related genes from the literature [28,29,30,31,32,33,34]. We performed an evaluation of the functional gene enrichment in the identified genes using a hypergeometric test. Figure 3 shows that the identified genes were statistically overrepresented in the compiled list of literature-reviewed genes with significant *p*-values. We found 75 genes that were enriched in a catalog of genes involved in lipid and intramuscular fat-related functions (*p*-value equal to 3.28 × 10^−4^), and 90 genes that were overrepresented in a list of mammary gland/milk-related genes (*p*-value equal to 1.51 × 10^−2^). These overrepresented genes are listed in Tables S4 and S5. This result indicates that the regions involved in the SNPs identified by the mutual information extractors include many functional genes that are closely associated with breed-specific characteristics. The functional analysis of these genes is detailed in the next section.

The identified SNPs that overlap the lipid/intramuscular fat or milk-related genes had dissimilar patterns in terms of heterozygosity in the Angus versus the Jersey breeds (Figures S4 and S5). Table 1 shows the frequencies of heterozygosity for the lipid/intramuscular fat and milk-related SNPs for the two breeds. The total average frequency of heterozygosity of the lipid/intramuscular fat-related SNPs in the Angus breed was 0.391. Interestingly, when one or more Jersey individuals exhibited heterozygous alleles at an SNP locus of these lipid/intramuscular fat genes, all the Angus individuals’ alleles at this SNP locus were homozygous (the number of lipid-intramuscular fat genes “with heterozygosity in Jersey” was 0 in the Angus breed). In contrast, if all the alleles of the lipid/intramuscular fat-related genes were homozygous in the Jersey breed, the frequency of heterozygosity at the same SNP position in the Angus breed reached 2.813. We also observed that the frequency of heterozygosity was 0.431 for the milk-related SNPs in Jersey. Similarly, if the alleles of the milk-related genes at an SNP locus were heterozygous in one or more Angus individuals, this heterozygosity did not occur at the same SNP locus in Jersey individuals (the number of milk-related genes in Jersey “with heterozygosity in Angus” was 0). Moreover, if all the alleles of the milk-related SNPs appeared to be homozygous in the Angus breed, the heterozygosity frequency at the same SNP position was 4.046 in the Jersey breed. As shown in Table 1, if the allele genotype of the identified SNPs was heterozygous in any individual of a breed, that heterozygosity did not occur in the same SNP of the other breed. We found large differences in the patterns of heterozygosity between the Angus and Jersey breeds, particularly for SNPs involved in breed-specific genes. 

### 3.4. Identification of Breed-Specific Genes 

We found discriminative SNPs based on conditional mutual information from the genomes of Angus and Jersey cattle with significant *p*-value levels. The candidate regions indicating strong associations between SNPs and phenotypic traits can be genetically important sites for Angus and Jersey selection. Moreover, these regions contain key genes associated with functional roles for beef and dairy production, and the identified genes were validated with a literature review and gene ontology (GO) analysis. 

Several of the identified genes were strongly associated with lipid metabolism for meat and milk production. FASN, LPL, and SCD, important lipogenic enzymes, have been reported to have an influence on lipid deposition, metabolism, and synthesis and are involved in the mammary regulation of milk fat synthesis [35,36,37]. In particular, SCD is a lipogenic enzyme responsible for influencing the fatty acid composition of muscle and adipose tissue, and the SCD genotype may be a marker for enhancing the nutritional quality of milk [38,39]. Genetic variations in INSIG1 are also related to the ratio of saturated to unsaturated fatty acids in milk, and the activity of INSIG1 affects cholesterol metabolism, lipogenesis, and glucose homeostasis in adipose tissue [39,40]. In addition, several studies have reported that GHR is a key gene influencing milk composition and yield and that polymorphisms in GHR are related to beef marbling [41,42]. Moreover, PPARGC1A is known to be a regulator of energy metabolism and controls the proliferation and differentiation of brown adipocytes [43]. The PPARGC1A gene has also been observed to play a role in the regulation of milk fat synthesis in dairy cattle [44]. 

In addition, we found genes involved in adipogenesis and adipose cellular functions. EBF1 has been reported to inhibit the differentiation of intramuscular adipocytes by increasing anti-adipogenic factors [43]. Recent studies indicated that FGFs (including FGF1 and FGF2) play a positive role in adipogenesis [43]. Specifically, FGF1 has pro-adipogenic activity on preadipocytes [45], and FGF2 induces the development and growth of adipose-tissue in muscles [46]. IGF1 also has distinct effects on preadipocytes and potentially on mature adipocytes [43,47]. In addition, MYOG regulates the formation of muscle myofibers, which are associated with meat production capacity and harbor several QTL for weight and marbling in cattle [37,48]. Moreover, TTN is one of the marker genes for marbling in beef, and polymorphisms in TTN are closely associated with myofibrillogenesis, which increases marbling levels [49].

Furthermore, we identified genes highly specialized for milk production and mammary gland-related processes. CSN1S1 and CSN3 are known to be key milk protein genes. These genes are closely related to milk yield parameters and milk quality, and many studies have reported that polymorphisms of casein genes influence milk composition and milk protein synthesis [50,51,52]. MFGE8 is specifically observed in the mammary glands of lactating mice and is overexpressed during lactation and associated with an increase in milk fat content [53]. In addition, GLYCAM1, a member of the mucin family, is a milk protein synthesized in the mammary gland that encodes a milk fat globule glycoprotein [54]. In addition to the genes responsible for dairy yield traits, we identified the gene for the KIT ligand, KITLG. Missense variations in KITLG influence the roan/white coat color in cattle [55]. KITLG is also known to be an attractive candidate gene for moderating coat color in pigs [56].

**Figure 4:**
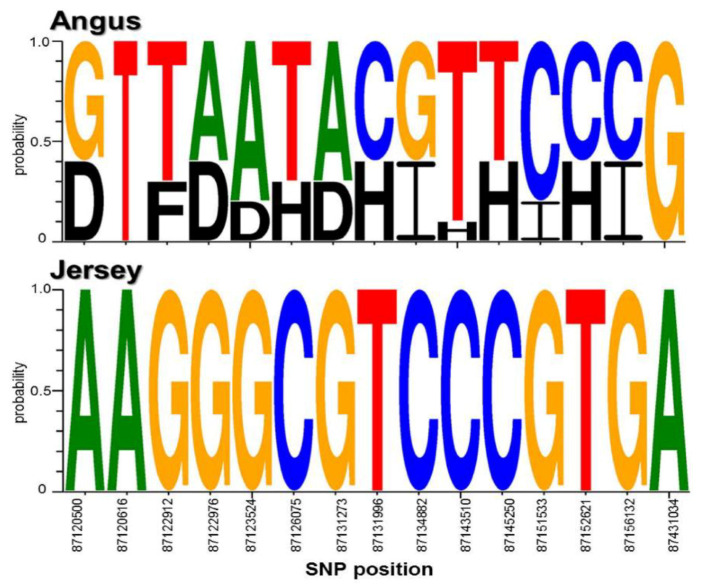
Genotypic profiles of the identified casein genes in Angus and Jersey. This is the genotype profiles of the SNPs in CSN1S1 and CSN3 in Angus and Jersey breeds. A, T, G, C, B, D, E, F, H, and I in the figure indicate AA, TT, GG, CC, AT/TA, AG/GA, AC/CA, TG/GT, TC/CT, and GC/CG genotypes, respectively.

In domesticated animals, research has supported the importance of the conservation of specific alleles or genotypes [57]. In particular, the widely conserved casein loci affect milk production and quality. Thus, we analyzed the genotype profiles of the found SNPs by our method in the identified casein genes, CSN1S1 and CSN3, in Angus and Jersey cattle. Interestingly, the result showed that the analyzed sequence logos clearly revealed different genotype profiles for each breed from the identified SNPs (Figure 4). The genome regions containing these genes were highly conserved, with less genetic variation observed in Jersey versus the Angus breed. 

### 3.5. Functional Enrichment Analysis of the Identified Genes 

**Figure 5:**
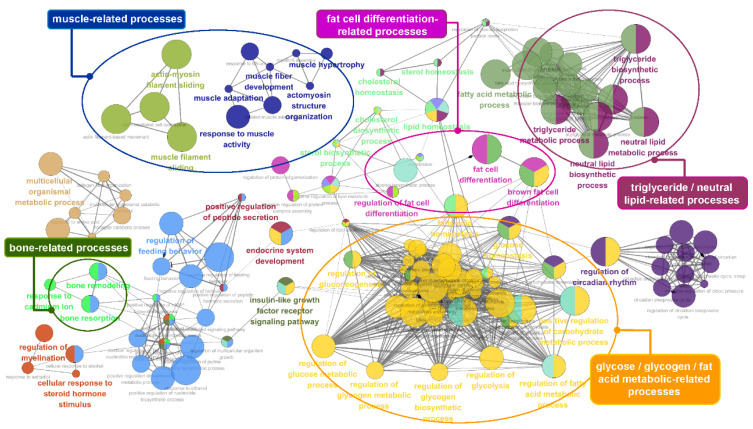
Gene ontology (GO) functional enrichment analysis of the 75 identified genes that overlapped with the lipid metabolism and intramuscular fat genes. The analyzed GO network consisted of distinct functional groups that are associated with energy- and lipid-related processes, such as triglyceride, fatty acid, and glucose metabolism as well as fat cell differentiation. The development of muscle- and bone-related processes is also annotated. The GO functionally grouped networks use terms as nodes (Bonferroni *p*-value < 0.05) and are linked according to their kappa score level (≥ 0.4). The size of the nodes corresponds to the statistical significance of the terms.

**Figure 6:**
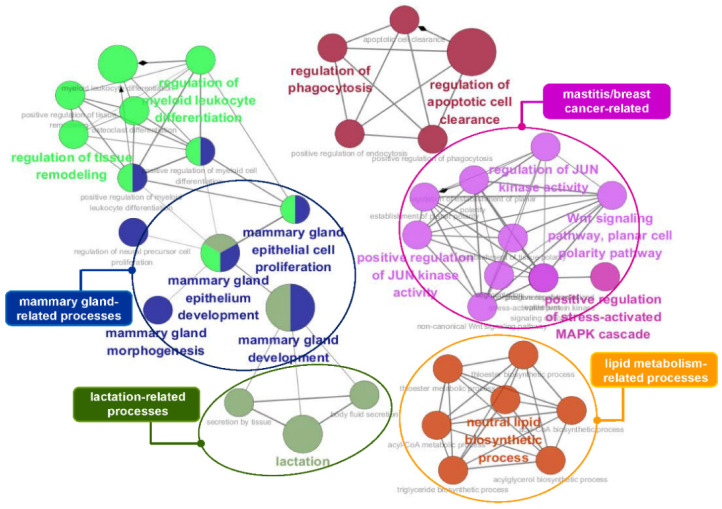
GO functional enrichment analysis of the 90 identified genes that overlapped with the mammary gland/milk production-related genes. This GO network comprised distinct functional groups that are implicated in mammary gland- and lactation-related processes. Moreover, several functional groups, such as Wnt signaling, the MAPK cascade, and JUN kinase activity, are associated with mastitis and breast cancer. GO functionally grouped networks use terms as nodes (Bonferroni *p*-value < 0.05) and are linked according to their kappa score level (≥0.4). The size of the nodes corresponds to the statistical significance of the terms.

To obtain insights into the biological processes involving the genes identified by conditional mutual information, we performed a functional analysis using ClueGO [58]. We identified functional genes that are useful for estimating the economic value of cattle, including 75 lipid and intramuscular fat-related genes and 90 milk production-related genes. Figure 5 and Figure 6 show the functional effects of these genes on biological processes. As shown in Figure 5, a large majority of the terms obtained by analyzing the 75 lipid and intramuscular fat-related genes were significantly associated with lipid and triglyceride metabolism, brown fat cell/fat cell differentiation, and energy metabolism, including the regulation of glucose, glycogen, and fatty acid metabolic processes. In particular, muscular and bone development-related terms were significantly enriched in our gene list and specifically included muscle adaptation, activity and hypertrophy, and bone remodeling. The core GO terms obtained by analyzing the 90 milk production-related genes were also related to mammary gland development, lactation, lipid metabolism, and mastitis-related processes, including JUN kinase activity regulation, MAPK kinase cascades, and the WNT signaling pathway (Figure 6). The details of the analyzed GO terms are described in Tables S6 and S7.

### 3.6. Distinct Genetic Variation on the Mitochondrial Genome

In this study, we identified eight SNPs (24.2% of the total SNPs on the mitochondrial genome) based on our mutual information extractors that act as genetic markers on the mitochondrial genome, discriminating between Angus and Jersey cattle. These SNPs included five genes in a total of 33 SNPs marked with a significant *p*-value (< 1.0 × 10^−4^) (Figure S6), and many of the included genes are implicated in energy mechanisms in cattle. 

Three of the five genes (ND1, ND2, and COX1) are associated with the development of intramuscular fat content in muscles. In detail, ND1 is highly expressed in oxidative muscles with higher intramuscular fat content [59], and ND2 is significantly correlated with marbling fat content in loin muscles [60]. COX is also strongly associated with triacylglycerol, a chief component of fat in muscles [61]. In addition, we analyzed the functional coherence of ND1, ND2, and COX1 with gene ontology analysis (Figure S7) [62]. Many of the overrepresented GO terms are closely associated with energy metabolism. The abundant GO terms include “generating energy for ATP synthesis”, “energy derivation through oxidation and respiratio”, “oxidative phosphorylation”, and “phosphorus metabolic processes.” The genotypes of the identified SNPs also showed clearly dissimilar patterns in the Angus versus the Jersey cattle (Figure S8). 

### 3.7. Analysis of the Overlapped Genetic Signatures Using Diverse Statistics

Combining different statistical methods can be more powerful than a single test to localize a source of selection if each statistic provides distinct information about the selective signatures [13]. We found putative genes showing genetic signatures that may have contributed to the development of Angus and Jersey-specific phenotypes by combining diverse statistics. We applied XP-CLR and XP-EHH tests to detect the putative selection genes by measuring changes in the allele frequency spectrum and the characteristics of extended haplotype homozygosity. In the results of the XP-CLR analysis, 230 and 203 putative selections genes were detected in Angus and Jersey, respectively, with the top 1% of their empirical distributions (empirical *p*-value < 1.0 × 10^−3^) (Table S8). Of these genes, 157 genes and 181 genes were shared among Angus and Jersey from MI and XP-CLR. The 226 Angus-selective genes and 253 Jersey-selective genes were detected with *p*-value < 1.0 × 10^−3^ using XP-EHH tests (Table S9). Of these 226 and 253 genes, 131 and 192 genes were found in common between MI and XP-EHH for Angus and Jersey, respectively. Finally, we observed 40 Angus-selective genes and 55 Jersey-selective genes at the intersection of MI, XP-CLR, and XP-EHH selection candidates, with the exception of various types of RNA, including 5S_rRNA, 7SK, U6, and so on (Tables S10 and S11). 

**Figure 7:**
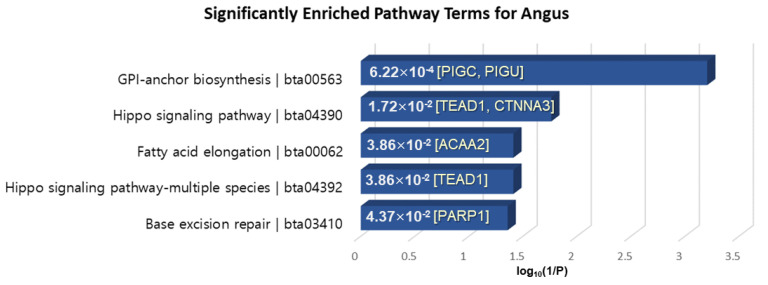
KEGG pathway enrichment analysis of the 40 overlapped genes in the Angus breed. The *x*-axis is the log10(1/*p*-value), and the annotated genes of the enriched pathways with significant *p*-values shown in bar graphs.

KEGG pathway analysis was performed by KOBAS 3.0, which is the latest web server for functional sets enrichment of genes [63]. The pathway analysis of 40 genes extracted in common among XP-CLR, XP-EHH and MI for Angus-selective genes showed significantly enriched terms “Glycosylphosphatidylinositol (GPI)-anchor biosynthesis”, “Hippo signaling pathway”, “Fatty acid elongation”, and “Base excision repair” with a *p*-value < 0.05 (Figure 7). PIGC, which exhibits fat depot-specific mRNA expression, is known to associate with lipid metabolism and obesity [64]. Moreover, ACAA2 is essential for de novo fatty acid synthesis and the activation of long-chain fatty acids and is expressed in the subcutaneous fat tissue of beef cattle involved in adipogenesis [65,66]. TEAD1 is well known as a mediator of skeletal muscle development, and transcriptional regulation of TEAD1 to muscle-specific genes is implemented in cooperation with numerous cofactors such as FoxO3a, which plays a key role in the muscle fiber types affecting meat color, meat tenderness and intramuscular fat content [67].

**Figure 8:**
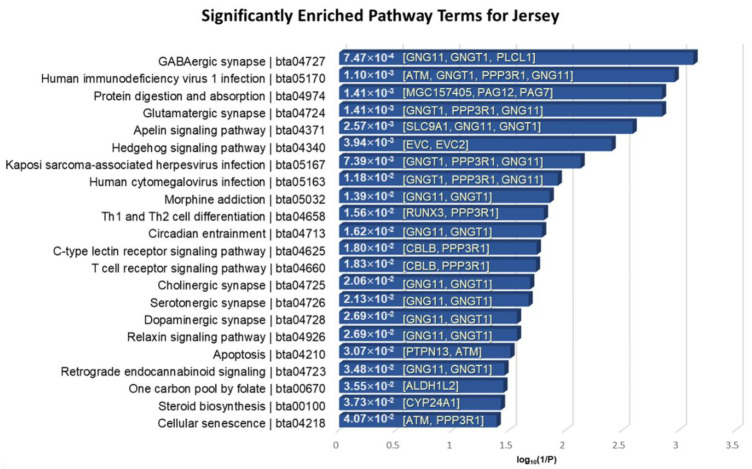
KEGG pathway enrichment analysis of the 55 overlapped genes in Jersey breed. The *x*-axis indicates the log10(1/*p*-value), and the annotated genes of the enriched pathways with significant *p*-values shown in bar graphs.

The 55 positively selected genes in Jersey compared to Angus were mainly involved in the nervous systems, immune systems, infectious diseases, signal transductions, environmental adaptation, endocrine systems, cell growth and death, and lipid metabolisms with a significant *p*-value < 0.05 (Figure 8). GNG11 and GNGT1 were significantly over-represented in several annotated pathways. GNG11 and GNGT1 code for G proteins, which function as key attributes of innate immune responses, and these are involved in functions relating to mastitis resistance [68]. In particular, PLCL1 encodes a protein that is involved in a component in the phospho-dependent endocytosis process of the GABA-A receptor. It is also located in CHR2: 86831095–87004473, of which the region is included in a specific trait of milk association QTL relating to milk fat percentage [69,70].

## 4. Discussion

Our study provides insights supporting the identification of discriminative SNPs with breed-specific genetic variations from the whole cattle genome. The used mutual information extractor explicitly found several genetic variations influencing beef and dairy traits in Angus and Jersey cattle from large-scale genome data. Genotype profiles using these phenotypic traits from the cattle genome can be analyzed to identify the key functional genes involved in the formation of breed characteristics.

In this study, we identified discriminative SNPs between Angus and Jersey breeds, and several analysis results based on the identified SNPs confirmed distinct differences between two cattle. The identified genes, including distinct SNPs, are associated with breed-specific functions, such as meat and milk production, respectively, and the contained SNPs showed clearly dissimilar genotypic patterns. Furthermore, several functional enrichment analyses revealed that distinct functional terms were enriched in the identified genes. 

Interestingly, the enriched GO terms in the fat-related genes were associated with lipid and energy metabolism, fat cell differentiation, and muscular/bone development-related terms, whereas the overrepresented terms in the milk-related genes were primarily involved in mammary gland development, lactation, and lipid metabolism. The lipid metabolism terms were enriched in both the fat- and milk-related genes because the traits for both meat and milk are closely associated with the lipid activity of cattle adipocytes [71]. These findings present that our approach can offer a new source of genetic variations influencing the breed-specific traits and show promise for advancing cattle genome research.

Moreover, we investigated the allele patterns of the identified SNPs associated with major functional genes. In particular, we found that casein genes clearly exhibited different breed-specific genotype profiles. The frequency of heterozygosity per SNP locus also showed clearly distinguishable patterns in Angus versus Jersey cattle. Interestingly, if the alleles of a candidate SNP are heterozygous in one specific breed, this heterozygosity does not occur in the same SNP locus of the other breed. This pattern indicates that genetic variation is associated with different properties depending on specific cattle breeds, which can influence the distinct traits of each individual. In addition, the distinct breed-specific allelic patterns of the candidate functional SNPs can provide insights for the discovery of new breed-specific hallmarks for discriminating between beef and milk cattle breeds. Furthermore, this approach allows us to understand the distinct genetic mechanisms underlying the formation of breed characteristics in domestic animals.

Despite numerous studies on the cattle genome, the details of the genetic variations in the mitochondrial genome of cattle have not been much explored relatively. However, mitochondria are important for metabolism, nutrition, and health in humans and animals. Several mitochondrial DNA mutants are reported in connection with a variety of complex traits, such as human disease, longevity, and so on [72,73]. Moreover, several studies have presented that mitochondrial genome polymorphisms in livestock are associated with economic traits, including meat quality, milk-yield, production, and reproduction [74,75,76,77]. In particular, considerable mitochondrial DNA diversity has been detected in dairy cattle, and the differences in mitochondrial DNA have been significantly related to milk-yield traits [78,79]. In this study, we found functional genomic regions with discriminative genetic variations between two cattle breeds on the mitochondrial genome. More interestingly, the contained SNP genotypes in the identified genes also showed explicitly different patterns in Angus versus Jersey cattle. Thus, the SNP positions identified on the mitochondrial genome are distinct regions with high discriminative capability, and their variation can be used to recognize genetic features for classifying the two breeds. Moreover, this finding can provide new clues for mitochondrial genome studies in cattle for economic traits.

Finally, our analysis focused on identifying the genetic variations distinguishing each cattle breed and representing the functional traits of cattle from genome sequence data, minimizing the use of genetic assumptions. To compare our method with other statistical methods, we performed an analysis of the selection signatures in the Angus and Jersey cattle using two approaches with different theoretical bases. Many statistical approaches, including F_ST_, iHS, XP-CLR, and XP-EHH, have been developed to detect the footprints left by selection in genomes. These methods rely on patterns of variation caused by the changes arising quickly in a population, such as allele frequency and haplotype length, to efficiently detect the genomic regions under selection. In addition, these methods use different time frames. In particular, XP-CLR, which utilizes changes in the phase of the allele frequency distribution between populations, has the power to identify older signatures compared with those based on extended linkage disequilibrium, such as XP-EHH [80].

In this study, we analyzed using XP-CLR and XP-EHH with conditional mutual information for providing meaningful results. The selection signature results are not completely consistent, but we found several candidate genomic regions. Frequently, combining different approaches can be more powerful than a single test if each statistic provides distinct information about the selective signatures [13]. Genetic regions revealed by XP-CLR and XP-EHH were putatively under positive selection, some of which could be crucial for understanding their unique properties. This is possible to produce larger lists of likely selective sweeps, and it may allow us to better understand how selection has effected the variations of a specific breed.

These statistical approaches are useful for detecting the genomic features that accompany the introduction of evolutionarily selective alleles in genome-wide studies, whereas our proposed method has the advantage of identifying potentially discriminative genetic variations in genome sequences. Our method can also assist with hypothesis formulation for genetic mechanisms in cattle, and thus, it provides a new approach for studying the distinct genomic regions related to breed-specific characteristics. Moreover, conditional mutual information can contribute to investigating the associations between distinct SNPs relevant to traits of interest and can considerably aid in understanding the evolution of cattle.

## 5. Conclusions

Our results described that beef and dairy cattle clearly show genetic differences at a genome-wide level. These implicate that the identified genes based on the extracted SNPs using conditional mutual information can contribute to discriminating the phenotypes of Angus and Jersey cattle, including beef and milk-yield traits. Moreover, the found SNPs showed that they can be involved in different molecular functions and mechanisms influencing the phenotypic differences between cattle breeds of distinct economic significance. Our analysis may provide potential genetic markers for the improvement in livestock productivity, and show the value of comparative genome study in cattle breeds based on an information-theoretic approach.

## Figures and Tables

**Table 1:** The average of the heterozygosity frequency for the lipid-intramuscular fat-related genes in Angus and milk-related genes in Jersey.

**Average of Heterozygosity Frequency of Lipid-Related Genes in Angus**	
Total heterozygosity frequency in Angus	0.391
With homozygosity in Jersey	2.813
With heterozygosity in Jersey	0
**Average of heterozygosity frequency of milk-related genes in Jersey**	
Total heterozygosity frequency in Jersey	0.431
With homozygosity in Angus	4.046
With heterozygosity in Angus	0

“Total average of heterozygosity frequency” is the average of the occurrence of heterozygosity per locus of all the SNPs in Angus and Jersey breed, respectively. “With homozygosity in Jersey (or Angus)” denotes the SNPs where all the individuals in a specific breed had homozygous alleles. In contrast, “With heterozygosity in Jersey (or Angus)” indicates the cases that the SNPs had at least one individual with the heterozygous allele in a breed.

## Supplementary Materials

The following are available online at https://www.mdpi.com/2073-4425/11/6/678/s1. Figure S1: Projection of 20 Angus and Jersey individuals plotted on the top two principal components, Figure S2: Ratio of the identified SNPs to all the SNPs on each chromosome and the number of genes, including the identified SNPs, Figure S3: Distribution of 858 SNPs for heterozygosity in the identified lipid/intramuscular fat-related genes in Angus versus Jersey cattle, Figure S4: Distribution of 852 SNPs for heterozygosity in the identified milk production-related genes in Angus versus Jersey cattle, Figure S5: Thirty-three SNPs on the mitochondrial genome plotted with negative log-scaled *p*-values, Figure S6: Significant GO terms for ND1, ND2, and COX1 on the mitochondrial genome, Figure S7: Genotype profiles for each SNP locus on the mitochondrial genome of Angus and Jersey breeds, Table S1: Summary of sequencing data, Table S2: Chromosomal distributions of the number of SNPs, Table S3: Chromosomal distributions of the number of SNPs identified by CMI, Table S4: The 75 identified lipid/intramuscular fat-related genes and the number of identified SNPs, Table S5: The 90 identified mammary gland/milk production-related genes and the number of identified SNPs, Table S6: Significant GO terms in the 75 identified lipid/intramuscular fat-related genes, Table S7: Significant GO terms in the 90 identified mammary gland/milk production-related genes, Table S8: Summary of XP-CLR, Table S9: Summary of XP-EHH, Table S10: Forty genes in the intersection of XP-CLR, XP-EHH, and conditional mutual information for Angus, Table S11: Fifty-five genes in the intersection of XP-CLR, XP-EHH, and conditional mutual information for Jersey.
