# Supplementary material for: Genome-Wide Identification of Discriminative Genetic Variations in Beef and Dairy Cattle via an Information-Theoretic Approach
Source: Genes (Basel). 2020 Jun 22;11(6):678. doi: 10.3390/genes11060678 (PMC7350245; doi:10.3390/genes11060678)
Supplement: Supplementary file 1 [file genes-11-00678-s001.zip › Suppl.Materials_revision.docx]

**Genome-wide identification of discriminative genetic variations in beef and dairy cattle via information-theoretic approach**

Authors: Soo-Jin Kim, Jung-Woo Ha, Heebal Kim*

| **Supplementary Tables and Figures** |
| --- |

**Supplementary Tables2-22**

Table S12

Table S23

Table S34

Table S45-7

Table S58-11

Table S612-16

Table S717-18

Table S1019-20

Table S1121-22

**Supplementary Figures23-30**

Figure S123

Figure S224

Figure S325

Figure S426

Figure S527

Figure S628

Figure S729

Figure S830

Table S1. Summary of sequencing data

| Sample ID | DNA Sequenced (bp) | Total Reads | Read Depth | Alignment Rate (%) | Genome Coverage (%) |
| --- | --- | --- | --- | --- | --- |
| 25 (Angus) | 36,469,506,744 | 368,378,856 | 13.71 | 97.10 | 99.00 |
| 41 (Angus) | 40,665,674,313 | 410,764,387 | 15.28 | 96.81 | 98.86 |
| 42 (Angus) | 39,278,606,342 | 404,934,086 | 14.76 | 97.08 | 98.99 |
| 45 (Angus) | 39,592,516,093 | 408,170,269 | 14.88 | 96.96 | 98.98 |
| 47 (Angus) | 39,068,627,668 | 398,659,466 | 14.68 | 96.86 | 98.93 |
| 48 (Angus) | 40,085,743,896 | 404,906,504 | 15.06 | 96.88 | 98.94 |
| 49 (Angus) | 39,389,755,829 | 406,079,957 | 14.80 | 97.00 | 98.82 |
| 50 (Angus) | 40,093,743,255 | 396,967,755 | 15.07 | 97.03 | 98.98 |
| 56 (Angus) | 38,068,812,536 | 376,918,936 | 14.31 | 97.56 | 98.82 |
| 60 (Angus) | 37,819,781,583 | 374,453,283 | 14.21 | 97.74 | 98.78 |
| 296 (Jersey) | 45,063,837,408 | 446,176,608 | 16.94 | 98.16 | 98.82 |
| 2040 (Jersey) | 51,245,773,496 | 507,383,896 | 19.26 | 98.37 | 98.71 |
| 3310 (Jersey) | 38,538,105,602 | 381,565,402 | 14.48 | 98.37 | 98.61 |
| 3344 (Jersey) | 40,238,521,503 | 398,401,203 | 15.12 | 98.31 | 98.79 |
| 3352 (Jersey) | 36,755,212,899 | 363,912,999 | 13.81 | 98.37 | 98.59 |
| 3369 (Jersey) | 44,567,697,330 | 441,264,330 | 16.75 | 98.48 | 98.66 |
| 3377 (Jersey) | 45,698,716,338 | 452,462,538 | 17.17 | 98.44 | 98.84 |
| 3454 (Jersey) | 48,051,823,832 | 475,760,632 | 18.06 | 98.62 | 98.70 |
| 4252 (Jersey) | 52,565,162,756 | 520,447,156 | 19.75 | 94.00 | 98.94 |
| 4718 (Jersey) | 46,875,378,256 | 464,112,656 | 17.62 | 98.57 | 98.68 |

Table S2. Chromosomal distributions of the number of SNPs

| Chromosome | Length | Changes | Change rate (%) |
| --- | --- | --- | --- |
| 1 | 158,337,067 | 847,638 | 0.535 |
| 2 | 137,060,424 | 674,521 | 0.492 |
| 3 | 121,430,405 | 591,758 | 0.487 |
| 4 | 120,829,699 | 644,727 | 0.534 |
| 5 | 121,191,424 | 623,495 | 0.514 |
| 6 | 119,458,736 | 668,648 | 0.560 |
| 7 | 112,638,659 | 558,879 | 0.496 |
| 8 | 113,384,836 | 562,437 | 0.496 |
| 9 | 105,708,250 | 534,506 | 0.506 |
| 10 | 104,305,016 | 555,881 | 0.533 |
| 11 | 107,310,763 | 535,464 | 0.499 |
| 12 | 91,163,125 | 553,374 | 0.607 |
| 13 | 84,240,350 | 406,813 | 0.483 |
| 14 | 84,648,390 | 444,191 | 0.525 |
| 15 | 85,296,676 | 469,098 | 0.550 |
| 16 | 81,724,687 | 448,153 | 0.548 |
| 17 | 75,158,596 | 427,134 | 0.568 |
| 18 | 66,004,023 | 339,426 | 0.514 |
| 19 | 64,057,457 | 324,511 | 0.507 |
| 20 | 72,042,655 | 387,805 | 0.538 |
| 21 | 71,599,096 | 374,502 | 0.523 |
| 22 | 61,435,874 | 294,449 | 0.479 |
| 23 | 52,530,062 | 359,628 | 0.685 |
| 24 | 62,714,930 | 337,488 | 0.538 |
| 25 | 42,904,170 | 246,318 | 0.574 |
| 26 | 51,681,464 | 288,123 | 0.557 |
| 27 | 45,407,902 | 259,844 | 0.572 |
| 28 | 46,312,546 | 260,308 | 0.562 |
| 29 | 51,505,224 | 317,591 | 0.617 |
| X | 148,823,899 | 325,067 | 0.218 |
| MT | 16,338 | 33 | 0.202 |
| Total | 2,660,922,743 | 13,661,810 | 0.513 |

Table S3. Chromosomal distributions of the number of SNPs identified by CMI

| Chromosome | Calling SNPs | Identified SNPs | Rate (%) |
| --- | --- | --- | --- |
| 1 | 847,638 | 8,436 | 0.995 |
| 2 | 674,521 | 7,679 | 1.138 |
| 3 | 591,758 | 5,414 | 0.915 |
| 4 | 644,727 | 5,106 | 0.792 |
| 5 | 623,495 | 5,662 | 0.908 |
| 6 | 668,648 | 5,409 | 0.809 |
| 7 | 558,879 | 8,735 | 1.563 |
| 8 | 562,437 | 4,151 | 0.738 |
| 9 | 534,506 | 4,368 | 0.817 |
| 10 | 555,881 | 4,127 | 0.742 |
| 11 | 535,464 | 5,122 | 0.957 |
| 12 | 553,374 | 3,673 | 0.664 |
| 13 | 406,813 | 7,769 | 1.910 |
| 14 | 444,191 | 2,870 | 0.646 |
| 15 | 469,098 | 6,081 | 1.296 |
| 16 | 448,153 | 2,319 | 0.517 |
| 17 | 427,134 | 3,194 | 0.748 |
| 18 | 339,426 | 2,617 | 0.771 |
| 19 | 324,511 | 3,947 | 1.216 |
| 20 | 387,805 | 4,504 | 1.161 |
| 21 | 374,502 | 1,876 | 0.501 |
| 22 | 294,449 | 3,131 | 1.063 |
| 23 | 359,628 | 2,438 | 0.678 |
| 24 | 337,488 | 2,015 | 0.597 |
| 25 | 246,318 | 741 | 0.301 |
| 26 | 288,123 | 2,492 | 0.865 |
| 27 | 259,844 | 2,771 | 1.066 |
| 28 | 260,308 | 3,197 | 1.228 |
| 29 | 317,591 | 2,785 | 0.877 |
| X | 325,067 | 3,913 | 24.242 |
| MT | 33 | 8 | 1.204 |
| Total | 13,661,810 | 126,550 | 0.926 |

Table S4. The 75 identified lipid/intramuscular fat-related genes and the number of identified SNPs.

| Ensemble Gene ID | Gene Symbol | Description | Location | #SNP |
| --- | --- | --- | --- | --- |
| ENSBTAG00000017218 | ACER3 | Bos taurus alkaline ceramidase 3 | 15: 57028596-57186215 | 4 |
| ENSBTAG00000001417 | ACSM1 | acyl-CoA synthetase medium-chain family member 1 | 25: 18349702-18413889 | 6 |
| ENSBTAG00000013303 | ACSS2 | Bos taurus acyl-CoA synthetase short-chain family member 2 | 13: 64794728-64841518 | 15 |
| ENSBTAG00000018694 | ACSS3 | Bos taurus acyl-CoA synthetase short-chain family member 3 | 5: 10731241-10905490 | 2 |
| ENSBTAG00000046332 | ACTA1 | Bos taurus actin, alpha 1, skeletal muscle | 28: 419154-421910 | 15 |
| ENSBTAG00000018255 | ACTN1 | Bos taurus actinin, alpha 1 | 10: 81023526-81121590 | 17 |
| ENSBTAG00000009696 | ACTN2 | Bos taurus actinin, alpha 2 | 28: 9403202-9450916 | 1 |
| ENSBTAG00000031188 | ADIG | Bos taurus adipogenin | 13: 68119517-68129193 | 1 |
| ENSBTAG00000019504 | ADRA1D | Bos taurus adrenergic, alpha-1D-, receptor | 13: 51370890-51392113 | 8 |
| ENSBTAG00000003764 | AKIRIN2 | Bos taurus akirin 2 | 9: 63127339-63150180 | 71 |
| ENSBTAG00000017121 | ALB | Bos taurus albumin | 6: 90232762-90251126 | 4 |
| ENSBTAG00000010123 | APOE | Bos taurus apolipoprotein E | 18: 53040105-53042792 | 1 |
| ENSBTAG00000038234 | BHMT2 | betaine--homocysteine S-methyltransferase 2 | 10: 10072118-10082600 | 9 |
| ENSBTAG00000002069 | BOLA | Bos taurus MHC class I heavy chain | 23: 28502524-28506312 | 1 |
| ENSBTAG00000012778 | CAPN2 | Bos taurus calpain 2 | 16: 27781671-27840009 | 9 |
| ENSBTAG00000005574 | CLU | Bos taurus clusterin | 8: 11043941-11061301 | 13 |
| ENSBTAG00000020056 | COL12A1 | collagen, type XII, alpha 1 | 9: 14869511-14983763 | 2 |
| ENSBTAG00000013472 | COL1A2 | Bos taurus collagen, type I, alpha 2 | 4: 11624470-11661163 | 52 |
| ENSBTAG00000021466 | COL3A1 | collagen alpha-1(III) chain precursor | 2: 7318227-7356937 | 9 |
| ENSBTAG00000006556 | COPB1 | Bos taurus coatomer protein complex, subunit beta 1 | 15: 38765050-38800769 | 9 |
| ENSBTAG00000043561 | COX1 | cytochrome c oxidase subunit I | MT: 5687-7231 | 2 |
| ENSBTAG00000033128 | CRH | Bos taurus corticotropin releasing hormone | 14: 32213577-32215193 | 9 |
| ENSBTAG00000011869 | CSRP3 | Bos taurus cysteine and glycine-rich protein 3 (cardiac LIM protein) | 29: 25994182-26014859 | 13 |
| ENSBTAG00000016420 | CTNNB1 | Bos taurus catenin (cadherin-associated protein), beta 1 | 22: 13842703-13889468 | 1 |
| ENSBTAG00000012442 | CATB | Bos taurus cathepsin B | 8: 7414945-7423429 | 11 |
| ENSBTAG00000012012 | CYB5A | Bos taurus cytochrome b5 type A | 24: 4413368-4445873 | 1 |
| ENSBTAG00000001154 | DGAT2 | Bos taurus diacylglycerol O-acyltransferase 2 | 15: 55940757-55973229 | 116 |
| ENSBTAG00000002497 | ELOVL2 | Bos taurus ELOVL fatty acid elongase 2 | 23: 45093668-45111322 | 1 |
| ENSBTAG00000015980 | FASN | fatty acid synthase | 19: 51384922-51403614 | 7 |
| ENSBTAG00000009733 | FBP1 | Bos taurus fructose-1,6-bisphosphatase 1 | 8: 82460864-82491694 | 20 |
| ENSBTAG00000001335 | GHR | Bos taurus growth hormone receptor | 20: 31890736-32199996 | 7 |
| ENSBTAG00000009770 | GPD2 | Bos taurus glycerol-3-phosphate dehydrogenase 2 | 2: 39765206-39912916 | 9 |
| ENSBTAG00000021516 | GSTA1 | Bos taurus glutathione S-transferase alpha 3 (GSTA3) | 23: 24875608-24890466 | 4 |
| ENSBTAG00000006354 | HP | Bos taurus haptoglobin | 18: 39173135-39179293 | 1 |
| ENSBTAG00000000087 | HSD17B12 | Bos taurus hydroxysteroid (17-beta) dehydrogenase 12 | 15: 74652043-74830690 | 21 |
| ENSBTAG00000020527 | IDH1 | Bos taurus isocitrate dehydrogenase 1 | 2: 96941049-96962496 | 2 |
| ENSBTAG00000011082 | IGF-I | Bos taurus insulin-like growth factor 1 | 5: 66532877-66604734 | 1 |
| ENSBTAG00000013003 | INS | Bos taurus insulin | 29: 50036101-50037262 | 1 |
| ENSBTAG00000001592 | INSIG1 | Bos taurus insulin induced gene 1 | 4: 117906798-117920021 | 2 |
| ENSBTAG00000021308 | IRS1 | insulin receptor substrate 1 | 2: 115790540-115794253 | 16 |
| ENSBTAG00000007689 | LPIN1 | lipin 1 | 11: 86051195-86128538 | 2 |
| ENSBTAG00000009165 | LPIN3 | lipin 3 | 13: 70666141-70683589 | 1 |
| ENSBTAG00000012855 | LPL | Bos taurus lipoprotein lipase | 8: 67481089-67511227 | 36 |
| ENSBTAG00000005333 | MB | Bos taurus myoglobin | 5: 74170471-74181260 | 4 |
| ENSBTAG00000019676 | MC4R | Bos taurus melanocortin 4 receptor | 24: 59670136-59671874 | 4 |
| ENSBTAG00000011808 | MSTN | Bos taurus myostatin | 2: 6213566-6220196 | 2 |
| ENSBTAG00000011392 | MYBPC1 | Bos taurus myosin binding protein C, slow type | 5: 65737956-65840833 | 26 |
| ENSBTAG00000007090 | MYH2 | Bos taurus myosin, heavy chain 2, skeletal muscle, adult | 19: 30137767-30165109 | 1 |
| ENSBTAG00000037794 | MYH4 | myosin, heavy chain 4, skeletal muscle | 19: 30080604-30103436 | 1 |
| ENSBTAG00000006030 | MYOG | Bos taurus myogenin (myogenic factor 4) | 16: 596920-599757 | 51 |
| ENSBTAG00000007930 | NCOA6 | nuclear receptor coactivator 6 | 13: 64639066-64726460 | 24 |
| ENSBTAG00000043558 | ND1 | NADH dehydrogenase subunit 1 | MT: 3101-4056 | 2 |
| ENSBTAG00000043571 | ND2 | NADH dehydrogenase subunit 2 | MT: 4266-5307 | 2 |
| ENSBTAG00000006907 | NEB | nebulin | 2: 44546002-44754701 | 5 |
| ENSBTAG00000004503 | NPY | Bos taurus neuropeptide Y | 4: 72068358-72075482 | 5 |
| ENSBTAG00000005218 | PDE3B | phosphodiesterase 3B, cGMP-inhibited | 15: 38466100-38626862 | 48 |
| ENSBTAG00000023429 | PLS1 | Bos taurus plastin 1 | 1: 127323050-127416649 | 16 |
| ENSBTAG00000009128 | POU1F1 | Bos taurus POU class 1 homeobox 1 | 1: 35009035-35024781 | 4 |
| ENSBTAG00000017024 | PPARGC1A | Bos taurus peroxisome proliferator-activated receptor gamma, coactivator 1 alpha | 6: 44854113-44960668 | 11 |
| ENSBTAG00000018747 | PRKAA2 | Bos taurus protein kinase, AMP-activated, alpha 2 catalytic subunit | 3: 90055299-90127142 | 1 |
| ENSBTAG00000007359 | PROP1 | Bos taurus PROP paired-like homeobox 1 | 7: 41205895-41209917 | 2 |
| ENSBTAG00000019080 | PTH | Bos taurus parathyroid hormone | 15: 39728332-39730868 | 3 |
| ENSBTAG00000012500 | RARA | retinoic acid receptor, alpha | 19: 41210900-41249293 | 4 |
| ENSBTAG00000013461 | RPL24 | Bos taurus ribosomal protein L24 | 1: 46415223-46420721 | 9 |
| ENSBTAG00000004742 | RUNX1 | runt-related transcription factor 1 | 1: 148678710-148773773 | 3 |
| ENSBTAG00000017339 | RUNX1T1 | Bos taurus runt-related transcription factor 1; translocated to, 1 (cyclin D-related) | 14: 74642806-74787356 | 45 |
| ENSBTAG00000005990 | S1PR1 | Bos taurus sphingosine-1-phosphate receptor 1 | 3: 42184097-42188752 | 3 |
| ENSBTAG00000045728 | SCD | Bos taurus stearoyl-CoA desaturase | 26: 21141592-21148318 | 1 |
| ENSBTAG00000004860 | SLC27A6 | Bos taurus solute carrier family 27 (fatty acid transporter), member 6 | 7: 26237929-26329594 | 7 |
| ENSBTAG00000009190 | SLC2A4 | Bos taurus solute carrier family 2 (facilitated glucose transporter), member 4 | 19: 27616793-27621455 | 15 |
| ENSBTAG00000003546 | TFAM | transcription factor A, mitochondrial | 28: 46067523-46079446 | 1 |
| ENSBTAG00000007823 | TG | Bos taurus thyroglobulin | 14: 9262251-9508938 | 2 |
| ENSBTAG00000005306 | TRH | Bos taurus thyrotropin-releasing hormone | 22: 56530332-56533155 | 2 |
| ENSBTAG00000026986 | TTN | titin | 2: 18054943-18329808 | 20 |
| ENSBTAG00000018170 | UTS2R | urotensin 2 receptor | 19: 50814750-50815904 | 2 |

Table S5. The 90 identified mammary gland/milk production-related genes and the number of identified SNPs.

| Ensemble Gene ID | Gene Symbol | Description | Location | #SNP |
| --- | --- | --- | --- | --- |
| ENSBTAG00000004281 | ACSS1 | Bos taurus acyl-CoA synthetase short-chain family member 1 | 13: 43029813-43073610 | 36 |
| ENSBTAG00000021933 | ALOX12 | arachidonate 12-lipoxygenase | 19: 27421125-27434258 | 2 |
| ENSBTAG00000023600 | APOD | apolipoprotein D | 1: 72670963-72684269 | 1 |
| ENSBTAG00000001057 | ARFGAP3 | Bos taurus ADP-ribosylation factor GTPase activating protein 3 | 5: 114291727-114341661 | 27 |
| ENSBTAG00000001305 | ATP2B2 | plasma membrane calcium-transporting ATPase 2 | 22: 55001027-55302080 | 21 |
| ENSBTAG00000015249 | B4GALT1 | Beta-1,4-galactosyltransferase 1 | 8: 76176882-76232330 | 2 |
| ENSBTAG00000013919 | BOLA-DRB3 | Bos taurus major histocompatibility complex, class II, DRB3 | 23: 25458594-25476944 | 50 |
| ENSBTAG00000018704 | BTG3 | Bos taurus BTG family, member 3 | 1: 18909512-18928469 | 1 |
| ENSBTAG00000017733 | CA2 | Bos taurus carbonic anhydrase II | 14: 79372712-79388600 | 1 |
| ENSBTAG00000037811 | CCL2 | Bos taurus chemokine | 19: 16232968-16234839 | 4 |
| ENSBTAG00000005269 | CCNB2 | Bos taurus cyclin B2 | 10: 51247755-51272733 | 1 |
| ENSBTAG00000031252 | CD82 | Bos taurus CD82 molecule | 15: 75507140-75567303 | 17 |
| ENSBTAG00000014764 | CD9 | Bos taurus CD9 molecule | 5: 104505691-104518399 | 9 |
| ENSBTAG00000012482 | CDCP1 | CUB domain containing protein 1 | 22: 54717553-54770691 | 1 |
| ENSBTAG00000005615 | CEACAM1 | Bos taurus carcinoembryonic antigen-related cell adhesion molecule 1 | 18: 51138290-51157109 | 1 |
| ENSBTAG00000011224 | CITED2 | Bos taurus Cbp/p300-interacting transactivator, with Glu/Asp-rich carboxy-terminal domain, 2 | 9: 78071006-78073397 | 58 |
| ENSBTAG00000005574 | CLU | Bos taurus clusterin | 8: 11043941-11061301 | 6 |
| ENSBTAG00000001710 | CMTM8 | Bos taurus CKLF-like MARVEL transmembrane domain containing 8 | 22: 6789385-6885301 | 13 |
| ENSBTAG00000040193 | COLQ | collagen-like tail subunit | 1: 154218714-154285721 | 5 |
| ENSBTAG00000002046 | COX8B | Bos taurus cytochrome c oxidase subunit VIII-H | 11: 107244220-107246853 | 1 |
| ENSBTAG00000007695 | CSN1S1 | Bos taurus casein alpha s1 | 6: 87141556-87159096 | 3 |
| ENSBTAG00000039787 | CSN3 | Bos taurus casein kappa | 6: 87378398-87392750 | 14 |
| ENSBTAG00000003397 | CTBP2 | Bos taurus C-terminal binding protein 2 | 26: 44888008-44936346 | 1 |
| ENSBTAG00000015566 | CTR9 | RNA polymerase-associated protein CTR9 homolog | 15: 42464354-42499295 | 3 |
| ENSBTAG00000011100 | CTSC | Bos taurus cathepsin C | 29: 7433482-7473315 | 2 |
| ENSBTAG00000004596 | CYP11B1 | Bos taurus cytochrome P450, subfamily XI B, polypeptide 1 | 14: 2705112-2710148 | 2 |
| ENSBTAG00000008329 | CYTIP | Bos taurus cytohesin 1 interacting protein | 2: 38682030-38711144 | 3 |
| ENSBTAG00000016152 | DAB2 | Dab, mitogen-responsive phosphoprotein, homolog 2 | 20: 35018908-35079162 | 2 |
| ENSBTAG00000003075 | DVL2 | dishevelled segment polarity protein 2 | 19: 27574027-27581405 | 2 |
| ENSBTAG00000017662 | EEF2K | eukaryotic elongation factor-2 kinase | 25: 20115008-20165902 | 8 |
| ENSBTAG00000016899 | MED8 | mediator complex subunit 8 | 3: 103212683-103217887 | 1 |
| ENSBTAG00000009813 | ELOVL1 | Bos taurus ELOVL fatty acid elongase 1 | 3: 103233547-103238303 | 2 |
| ENSBTAG00000003607 | FAM20C | family with sequence similarity 20, member C | 25: 42694393-42728472 | 8 |
| ENSBTAG00000007976 | FAM3C | Bos taurus family with sequence similarity 3, member C | 4: 86620370-86674480 | 1 |
| ENSBTAG00000046509 | FAM46C | family with sequence similarity 46, member C | 3: 25703050-25705109 | 1 |
| ENSBTAG00000015980 | FASN | fatty acid synthase | 19: 51384922-51403614 | 7 |
| ENSBTAG00000005691 | FGF2 | Bos taurus fibroblast growth factor 2 | 17: 35199615-35259135 | 1 |
| ENSBTAG00000021581 | FHOD3 | formin homology 2 domain containing 3 | 24: 20649326-21172169 | 2 |
| ENSBTAG00000003440 | FRRS1 | Bos taurus ferric-chelate reductase 1 | 3: 43651196-43676797 | 2 |
| ENSBTAG00000001335 | GHR | Bos taurus growth hormone receptor | 20: 31890736-32199996 | 7 |
| ENSBTAG00000008124 | GK | Bos taurus glycerol kinase | X: 118112447-118186827 | 1 |
| ENSBTAG00000038186 | GLRX | Bos taurus glutaredoxin | 7: 97531726-97541600 | 10 |
| ENSBTAG00000013417 | GLYCAM1 | Bos taurus glycosylation-dependent cell adhesion molecule 1 | 5: 25595262-25598162 | 2 |
| ENSBTAG00000002767 | GNB4 | Bos taurus guanine nucleotide binding protein | 1: 88225885-88257992 | 2 |
| ENSBTAG00000019317 | GPS1 | Bos taurus G protein pathway suppressor 1 | 19: 51422926-51427197 | 1 |
| ENSBTAG00000038748 | HBB | Bos taurus hemoglobin, beta | 15: 49022978-49024619 | 2 |
| ENSBTAG00000012586 | HSPD1 | heat shock 60kDa protein 1 (chaperonin) | 2: 86438979-86449372 | 3 |
| ENSBTAG00000021187 | ID2 | Bos taurus inhibitor of DNA binding 2, dominant negative helix-loop-helix protein | 11: 88584862-88587166 | 1 |
| ENSBTAG00000001592 | INSIG1 | Bos taurus insulin induced gene 1 | 4: 117906798-117920021 | 2 |
| ENSBTAG00000012083 | IRX3 | Bos taurus iroquois homeobox 3 | 18: 22692066-22694550 | 5 |
| ENSBTAG00000019929 | ITGAV | integrin, alpha V | 2: 9651631-9760100 | 1 |
| ENSBTAG00000004515 | KCNK1 | Bos taurus potassium channel, subfamily K, member 1 | 28: 6492389-6559855 | 23 |
| ENSBTAG00000009383 | KIF11 | Bos taurus kinesin family member 11 | 26: 14042393-14087768 | 1 |
| ENSBTAG00000005859 | LALBA | Bos taurus lactalbumin, alpha- | 5: 31347861-31349882 | 11 |
| ENSBTAG00000003636 | LIPA | lipase A, lysosomal acid, cholesterol esterase | 26: 10988284-11030717 | 1 |
| ENSBTAG00000012855 | LPL | Bos taurus lipoprotein lipase | 8: 67481089-67511227 | 36 |
| ENSBTAG00000003300 | MFGE8 | milk fat globule-EGF factor 8 protein | 21: 20889913-20904968 | 1 |
| ENSBTAG00000004952 | MFSD4 | major facilitator superfamily domain containing 4 | 16: 3168635-3193846 | 1 |
| ENSBTAG00000012370 | MGP | Bos taurus matrix Gla protein | 5: 95456444-95459983 | 1 |
| ENSBTAG00000017104 | MUC1 | Bos taurus mucin 1, cell surface associated | 3: 15482856-15486936 | 3 |
| ENSBTAG00000004651 | NME1 | Bos taurus non-metastatic cells 1, protein (NM23A) expressed in (NME1) | 19: 36223570-36225626 | 1 |
| ENSBTAG00000016525 | ITGA1 | integrin, alpha 1 | 20: 26116747-26227530 | 3 |
| ENSBTAG00000003268 | PELO | Bos taurus pelota homolog | 20: 26278617-26280571 | 142 |
| ENSBTAG00000034985 | PHLDA1 | Bos taurus pleckstrin homology-like domain, family A, member 1 | 5: 5557939-5559917 | 1 |
| ENSBTAG00000015230 | PLA2G12A | Bos taurus phospholipase A2, group XIIA | 6: 16828642-16843001 | 3 |
| ENSBTAG00000009128 | POU1F1 | Bos taurus POU class 1 homeobox 1 | 1: 35009035-35024781 | 4 |
| ENSBTAG00000017024 | PPARGC1A | Bos taurus peroxisome proliferator-activated receptor gamma, coactivator 1 alpha | 6: 44854113-44960668 | 11 |
| ENSBTAG00000006538 | PTHLH | Bos taurus parathyroid hormone-like hormone | 5: 82246522-82258858 | 16 |
| ENSBTAG00000005835 | PYCR2 | Bos taurus pyrroline-5-carboxylate reductase family, member 2 | 16: 29695734-29699696 | 7 |
| ENSBTAG00000009871 | RAB18 | Bos taurus RAB18, member RAS oncogene family | 13: 37303894-37331999 | 9 |
| ENSBTAG00000031962 | RAB20 | RAB20, member RAS oncogene family | 12: 89182100-89212365 | 1 |
| ENSBTAG00000003658 | RELN | Bos taurus reelin | 4: 44892394-45289293 | 4 |
| ENSBTAG00000018691 | RHOU | Bos taurus ras homolog gene family, member U | 28: 2167764-2177307 | 14 |
| ENSBTAG00000015904 | RORA | RAR-related orphan receptor A | 10: 48949618-49750993 | 75 |
| ENSBTAG00000045728 | SCD | Bos taurus stearoyl-CoA desaturase | 26: 21141592-21148318 | 1 |
| ENSBTAG00000005668 | SLC39A8 | Bos taurus solute carrier family 39 | 6: 23816193-23898886 | 16 |
| ENSBTAG00000010357 | ST6GAL1 | Bos taurus ST6 beta-galactosamide alpha-2,6-sialyltranferase 1 | 1: 80788014-80830464 | 20 |
| ENSBTAG00000013761 | STMN1 | Bos taurus stathmin 1 | 2: 127773927-127779431 | 11 |
| ENSBTAG00000002065 | TFAP2C | Bos taurus transcription factor AP-2 gamma | 13: 59903160-59912715 | 1 |
| ENSBTAG00000008924 | TNFSF11 | Bos taurus tumor necrosis factor (ligand) superfamily, member 11 | 12: 12741069-12782474 | 19 |
| ENSBTAG00000032884 | TNP2 | Bos taurus transition protein 2 | 25: 9988822-9990357 | 3 |
| ENSBTAG00000001444 | TNXB | tenascin-X precursor | 23: 27085415-27136954 | 5 |
| ENSBTAG00000002837 | TOR1B | torsin family 1, member B | 11: 100210927-100215693 | 2 |
| ENSBTAG00000001069 | TP53 | Bos taurus tumor protein p53 | 19: 27985495-27997841 | 10 |
| ENSBTAG00000003336 | TPD52L1 | Bos taurus tumor protein D52-like 1 | 9: 26310580-26403759 | 2 |
| ENSBTAG00000007562 | TRAM1 | translocation associated membrane protein 1 | 14: 36385096-36417387 | 1 |
| ENSBTAG00000027246 | UBD | Bos taurus ubiquitin D | 23: 28918428-28920742 | 1 |
| ENSBTAG00000044150 | UCK2 | Bos taurus uridine-cytidine kinase 2 | 3: 3045781-3119590 | 2 |
| ENSBTAG00000018517 | VLDLR | Bos taurus very low density lipoprotein receptor | 8: 42109679-42141155 | 28 |
| ENSBTAG00000012519 | XDH | Bos taurus xanthine dehydrogenase | 11: 14176298-14281717 | 2 |

Table S6. Significant GO terms in the 75 identified lipid/intramuscular fat-related genes.

| GO:ID | GO terms | *P*-value | Group | Associated genes |
| --- | --- | --- | --- | --- |
| GO:0035383 | thioester metabolic process | 1.8e-03 | Group0 | ACSS2, DGAT2, ELOVL2, FASN, HSD17B12 |
| GO:0006637 | acyl-CoA metabolic process | 1.8e-03 | Group0 | ACSS2, DGAT2, ELOVL2, FASN, HSD17B12 |
| GO:0019432 | triglyceride biosynthetic process | 2.2e-05 | Group0 | DGAT2, ELOVL2, FASN, HSD17B12, LPIN1, LPL |
| GO:0035384 | thioester biosynthetic process | 2.4e-03 | Group0 | ACSS2, ELOVL2, FASN, HSD17B12 |
| GO:0071616 | acyl-CoA biosynthetic process | 2.4e-03 | Group0 | ACSS2, ELOVL2, FASN, HSD17B12 |
| GO:0006633 | fatty acid biosynthetic process | 2.5e-06 | Group0 | ACSM1, ACSS2, ELOVL2, FASN, HSD17B12, INSIG1, LPL, PRKAA2, SCD |
| GO:0006631 | fatty acid metabolic process | 2.6e-10 | Group0 | ACSM1, ACSS2, ELOVL2, FASN, GHR, HSD17B12, INS, INSIG1, IRS1, LPIN1, LPIN3, LPL, PPARGC1A, PRKAA2, SCD, SLC27A6 |
| GO:0046460 | neutral lipid biosynthetic process | 2.7e-05 | Group0 | DGAT2, ELOVL2, FASN, HSD17B12, LPIN1, LPL |
| GO:0046463 | acylglycerol biosynthetic process | 2.7e-05 | Group0 | DGAT2, ELOVL2, FASN, HSD17B12, LPIN1, LPL |
| GO:0035336 | long-chain fatty-acyl-CoA metabolic process | 3.1e-04 | Group0 | DGAT2, ELOVL2, FASN, HSD17B12 |
| GO:0006641 | triglyceride metabolic process | 4.5e-06 | Group0 | APOE, DGAT2, ELOVL2, FASN, HSD17B12, INSIG1, LPIN1, LPL |
| GO:0072330 | monocarboxylic acid biosynthetic process | 4.8e-05 | Group0 | ACSM1, ACSS2, ELOVL2, FASN, HSD17B12, INSIG1, LPL, PRKAA2, SCD |
| GO:0035337 | fatty-acyl-CoA metabolic process | 5.1e-04 | Group0 | DGAT2, ELOVL2, FASN, HSD17B12 |
| GO:0035338 | long-chain fatty-acyl-CoA biosynthetic process | 5.6e-03 | Group0 | ELOVL2, FASN, HSD17B12 |
| GO:0046949 | fatty-acyl-CoA biosynthetic process | 6.7e-03 | Group0 | ELOVL2, FASN, HSD17B12 |
| GO:0006639 | acylglycerol metabolic process | 6.7e-06 | Group0 | APOE, DGAT2, ELOVL2, FASN, HSD17B12, INSIG1, LPIN1, LPL |
| GO:0006638 | neutral lipid metabolic process | 7.1e-06 | Group0 | APOE, DGAT2, ELOVL2, FASN, HSD17B12, INSIG1, LPIN1, LPL |
| GO:0046686 | response to cadmium ion | 9.4e-03 | Group1 | CTNNB1, CYB5A, PTH |
| GO:0045453 | bone resorption | 1.1e-04 | Group1 | CTNNB1, MC4R, PTH, S1PR1 |
| GO:0046849 | bone remodeling | 4.1e-03 | Group1 | CTNNB1, MC4R, PTH, S1PR1 |
| GO:0045913 | positive regulation of carbohydrate metabolic process | 1.4e-05 | Group2 | INS, IRS1, POU1F1, PPARGC1A, PRKAA2, PTH |
| GO:0010676 | positive regulation of cellular carbohydrate metabolic process | 5.4e-06 | Group2 | INS, IRS1, POU1F1, PPARGC1A, PRKAA2, PTH |
| GO:0043255 | regulation of carbohydrate biosynthetic process | 9.7e-05 | Group2 | FBP1, INS, IRS1, POU1F1, PPARGC1A, PTH |
| GO:0033500 | carbohydrate homeostasis | 1.2e-03 | Group3 | CRH, INS, IRS1, PDE3B, PPARGC1A, PRKAA2, SLC2A4 |
| GO:0042593 | glucose homeostasis | 1.2e-03 | Group3 | CRH, INS, IRS1, PDE3B, PPARGC1A, PRKAA2, SLC2A4 |
| GO:0006111 | regulation of gluconeogenesis | 3.2e-03 | Group3 | FBP1, INS, PPARGC1A |
| GO:0050873 | brown fat cell differentiation | 4.0e-05 | Group3 | ADIG, INS, MB, PPARGC1A, SLC2A4 |
| GO:0045444 | fat cell differentiation | 7.3e-05 | Group3 | ADIG, INS, INSIG1, LPIN1, MB, PPARGC1A, RUNX1T1, SLC2A4 |
| GO:0006695 | cholesterol biosynthetic process | 1.4e-03 | Group4 | APOE, INSIG1, PRKAA2 |
| GO:0055089 | fatty acid homeostasis | 1.8e-03 | Group4 | DGAT2, INS, PRKAA2 |
| GO:0042632 | cholesterol homeostasis | 3.3e-03 | Group4 | ACSM1, APOE, DGAT2 |
| GO:0055088 | lipid homeostasis | 7.5e-04 | Group4 | ACSM1, APOE, DGAT2, INS, LPL, PRKAA2 |
| GO:0044275 | cellular carbohydrate catabolic process | 1.1e-03 | Group5 | FBP1, GPD2, INS, PPARGC1A, PRKAA2 |
| GO:0033500 | carbohydrate homeostasis | 1.2e-03 | Group5 | CRH, INS, IRS1, PDE3B, PPARGC1A, PRKAA2, SLC2A4 |
| GO:0042593 | glucose homeostasis | 1.2e-03 | Group5 | CRH, INS, IRS1, PDE3B, PPARGC1A, PRKAA2, SLC2A4 |
| GO:0046323 | glucose import | 1.3e-03 | Group5 | INS, IRS1, PTH, SLC2A4 |
| GO:0043470 | regulation of carbohydrate catabolic process | 1.3e-03 | Group5 | FBP1, INS, PPARGC1A, PRKAA2 |
| GO:0043471 | regulation of cellular carbohydrate catabolic process | 1.3e-03 | Group5 | FBP1, INS, PPARGC1A, PRKAA2 |
| GO:0009250 | glucan biosynthetic process | 1.4e-03 | Group5 | INS, IRS1, PTH |
| GO:0005978 | glycogen biosynthetic process | 1.4e-03 | Group5 | INS, IRS1, PTH |
| GO:0006695 | cholesterol biosynthetic process | 1.4e-03 | Group5 | APOE, INSIG1, PRKAA2 |
| GO:0045913 | positive regulation of carbohydrate metabolic process | 1.4e-05 | Group5 | INS, IRS1, POU1F1, PPARGC1A, PRKAA2, PTH |
| GO:0055089 | fatty acid homeostasis | 1.8e-03 | Group5 | DGAT2, INS, PRKAA2 |
| GO:0010675 | regulation of cellular carbohydrate metabolic process | 2.0e-04 | Group5 | FBP1, INS, IRS1, POU1F1, PPARGC1A, PRKAA2, PTH |
| GO:0042752 | regulation of circadian rhythm | 2.1e-04 | Group5 | ALB, CRH, PPARGC1A, PRKAA2, UTS2R |
| GO:0010962 | regulation of glucan biosynthetic process | 2.9e-03 | Group5 | INS, IRS1, PTH |
| GO:0005979 | regulation of glycogen biosynthetic process | 2.9e-03 | Group5 | INS, IRS1, PTH |
| GO:0006109 | regulation of carbohydrate metabolic process | 2.9e-04 | Group5 | FBP1, INS, IRS1, POU1F1, PPARGC1A, PRKAA2, PTH |
| GO:0019217 | regulation of fatty acid metabolic process | 3.1e-03 | Group5 | INS, INSIG1, IRS1, PPARGC1A, PRKAA2 |
| GO:0006111 | regulation of gluconeogenesis | 3.2e-03 | Group5 | FBP1, INS, PPARGC1A |
| GO:0045725 | positive regulation of glycogen biosynthetic process | 3.7e-03 | Group5 | INS, IRS1, PTH |
| GO:0050873 | brown fat cell differentiation | 4.0e-05 | Group5 | ADIG, INS, MB, PPARGC1A, SLC2A4 |
| GO:0010907 | positive regulation of glucose metabolic process | 4.0e-05 | Group5 | INS, IRS1, PPARGC1A, PRKAA2, PTH |
| GO:0070873 | regulation of glycogen metabolic process | 4.2e-03 | Group5 | INS, IRS1, PTH |
| GO:0035270 | endocrine system development | 4.5e-03 | Group5 | CRH, INS, IRS1, PDE3B, POU1F1, PROP1, TG, TRH |
| GO:0010676 | positive regulation of cellular carbohydrate metabolic process | 5.4e-06 | Group5 | INS, IRS1, POU1F1, PPARGC1A, PRKAA2, PTH |
| GO:0006094 | gluconeogenesis | 5.5e-03 | Group5 | FBP1, GPD2, INS, PPARGC1A |
| GO:0070875 | positive regulation of glycogen metabolic process | 5.6e-03 | Group5 | INS, IRS1, PTH |
| GO:0006110 | regulation of glycolysis | 6.0e-04 | Group5 | FBP1, INS, PPARGC1A, PRKAA2 |
| GO:0010906 | regulation of glucose metabolic process | 6.3e-04 | Group5 | FBP1, INS, IRS1, PPARGC1A, PRKAA2, PTH |
| GO:0043467 | regulation of generation of precursor metabolites and energy | 6.3e-05 | Group5 | FBP1, INS, IRS1, PPARGC1A, PRKAA2, PTH |
| GO:0055088 | lipid homeostasis | 7.5e-04 | Group5 | ACSM1, APOE, DGAT2, INS, LPL, PRKAA2 |
| GO:0043255 | regulation of carbohydrate biosynthetic process | 9.7e-05 | Group5 | FBP1, INS, IRS1, POU1F1, PPARGC1A, PTH |
| GO:0070252 | actin-mediated cell contraction | 1.2e-05 | Group6 | ACTA1, ACTN2, MYBPC1, MYH2, MYH4, NEB, TTN |
| GO:0033275 | actin-myosin filament sliding | 3.7e-08 | Group6 | ACTA1, ACTN2, MYBPC1, MYH2, MYH4, NEB, TTN |
| GO:0030049 | muscle filament sliding | 3.7e-08 | Group6 | ACTA1, ACTN2, MYBPC1, MYH2, MYH4, NEB, TTN |
| GO:0030048 | actin filament-based movement | 4.1e-05 | Group6 | ACTA1, ACTN2, MYBPC1, MYH2, MYH4, NEB, TTN |
| GO:0044236 | multicellular organismal metabolic process | 3.1e-03 | Group7 | COL12A1, COL1A2, COL3A1, CTSB, GHR, MC4R |
| GO:0071229 | cellular response to acid | 4.3e-03 | Group7 | CAPN2, COL1A2, COL3A1, DGAT2, PPARGC1A, RARA |
| GO:0030199 | collagen fibril organization | 8.7e-03 | Group7 | COL12A1, COL1A2, COL3A1 |
| GO:0045471 | response to ethanol | 1.0e-04 | Group8 | APOE, CRH, MSTN, PTH, RARA, SLC2A4, TRH |
| GO:0045453 | bone resorption | 1.1e-04 | Group8 | CTNNB1, MC4R, PTH, S1PR1 |
| GO:2000252 | negative regulation of feeding behavior | 2.9e-04 | Group8 | INS, MC4R, TRH |
| GO:0048521 | negative regulation of behavior | 3.3e-03 | Group8 | CRH, INS, MC4R, TRH |
| GO:0002793 | positive regulation of peptide secretion | 3.5e-03 | Group8 | CRH, INS, TRH |
| GO:0060259 | regulation of feeding behavior | 3.7e-03 | Group8 | INS, MC4R, TRH |
| GO:0030810 | positive regulation of nucleotide biosynthetic process | 3.7e-03 | Group8 | APOE, CRH, MC4R, PPARGC1A, PTH |
| GO:1900373 | positive regulation of purine nucleotide biosynthetic process | 3.7e-03 | Group8 | APOE, CRH, MC4R, PPARGC1A, PTH |
| GO:0046849 | bone remodeling | 4.1e-03 | Group8 | CTNNB1, MC4R, PTH, S1PR1 |
| GO:0035270 | endocrine system development | 4.5e-03 | Group8 | CRH, INS, IRS1, PDE3B, POU1F1, PROP1, TG, TRH |
| GO:0009755 | hormone-0mediated signaling pathway | 5.2e-03 | Group8 | CRH, GHR, PTH, TRH |
| GO:0045981 | positive regulation of nucleotide metabolic process | 6.4e-03 | Group8 | APOE, CRH, MC4R, PPARGC1A, PTH |
| GO:1900544 | positive regulation of purine nucleotide metabolic process | 6.4e-03 | Group8 | APOE, CRH, MC4R, PPARGC1A, PTH |
| GO:0007631 | feeding behavior | 9.9e-03 | Group8 | CRH, INS, MC4R, NPY, TRH |
| GO:0046460 | neutral lipid biosynthetic process | 2.7e-05 | Group9 | DGAT2,ELOVL2, FASN, HSD17B12, LPIN1, LPL |
| GO:0046463 | acylglycerol biosynthetic process | 2.7e-05 | Group9 | DGAT2,ELOVL2, FASN, HSD17B12, LPIN1, LPL |
| GO:0006641 | triglyceride metabolic process | 4.5e-06 | Group9 | APOE, DGAT2,ELOVL2, FASN, HSD17B12, INSIG1, LPIN1, LPL |
| GO:0006639 | acylglycerol metabolic process | 6.7e-06 | Group9 | APOE, DGAT2,ELOVL2, FASN, HSD17B12, INSIG1, LPIN1, LPL |
| GO:0006638 | neutral lipid metabolic process | 7.1e-06 | Group9 | APOE, DGAT2,ELOVL2, FASN, HSD17B12, INSIG1, LPIN1, LPL |
| GO:0055088 | lipid homeostasis | 7.5e-04 | Group9 | ACSM1, APOE, DGAT2, INS, LPL, PRKAA2 |
| GO:0019217 | regulation of fatty acid metabolic process | 3.1e-03 | Group10 | INS, INSIG1, IRS1, PPARGC1A, PRKAA2 |
| GO:0046165 | alcohol biosynthetic process | 6.2e-04 | Group10 | ACER3, APOE, INSIG1, LPIN1, LPIN3, POU1F1, PRKAA2 |
| GO:0055088 | lipid homeostasis | 7.5e-04 | Group10 | ACSM1, APOE, DGAT2, INS, LPL, PRKAA2 |
| GO:0030239 | myofibril assembly | 1.2e-03 | Group11 | ACTA1, CSRP3, TTN |
| GO:0014850 | response to muscle activity | 2.4e-03 | Group11 | MSTN, MYOG, PPARGC1A |
| GO:0043500 | muscle adaptation | 2.8e-03 | Group11 | ACTA1, MSTN, MYOG |
| GO:0031032 | actomyosin structure organization | 2.9e-03 | Group11 | ACTA1, CSRP3, TTN |
| GO:0048747 | muscle fiber development | 3.3e-03 | Group11 | ACTA1, MYOG, TTN |
| GO:0014888 | striated muscle adaptation | 3.5e-03 | Group11 | ACTA1, MSTN, MYOG |
| GO:0014896 | muscle hypertrophy | 8.7e-03 | Group11 | CSRP3, MSTN, TTN |
| GO:0050873 | brown fat cell differentiation | 4.0e-05 | Group12 | ADIG, INS, MB, PPARGC1A, SLC2A4 |
| GO:0045598 | regulation of fat cell differentiation | 5.0e-03 | Group12 | ADIG, INS, INSIG1, LPIN1 |
| GO:0045444 | fat cell differentiation | 7.3e-05 | Group12 | ADIG, INS, INSIG1, LPIN1, MB, PPARGC1A, RUNX1T1, SLC2A4 |
| GO:0071383 | cellular response to steroid hormone stimulus | 3.1e-03 | Group13 | APOE, CRH, CTNNB1, MYOG |
| GO:0031641 | regulation of myelination | 1.2e-03 | Group13 | CTNNB1, RARA, TG |
| GO:0002576 | platelet degranulation | 9.4e-03 | Group14 | ACTN1, ACTN2, ALB, CLU, TTN |
| GO:0002793 | positive regulation of peptide secretion | 3.5e-03 | Group15 | CRH, INS, TRH |
| GO:0035270 | endocrine system development | 4.5e-03 | Group15 | CRH, INS, PDE3B, POU1F1, PROP1, TG |
| GO:0055088 | lipid homeostasis | 7.5e-04 | Group17 | ACSM1, APOE, DGAT2, INS, LPL, PRKAA2 |
| GO:0033500 | carbohydrate homeostasis | 1.2e-03 | Group18 | CRH, INS, IRS1, PDE3B, PPARGC1A, PRKAA2, SLC2A4 |
| GO:0042593 | glucose homeostasis | 1.2e-03 | Group18 | CRH, INS, IRS1, PDE3B, PPARGC1A, PRKAA2, SLC2A4 |
| GO:0042752 | regulation of circadian rhythm | 2.1e-04 | Group18 | ALB, CRH, PPARGC1A, PRKAA2, UTS2R |
| GO:0042753 | positive regulation of circadian rhythm | 3.0e-03 | Group18 | ALB, CRH, UTS2R |
| GO:0042749 | regulation of circadian sleep/wake cycle | 3.7e-03 | Group18 | ALB, CRH, UTS2R |
| GO:0045187 | regulation of circadian sleep/wake cycle | 3.7e-03 | Group18 | ALB, CRH, UTS2R |
| GO:0050802 | circadian sleep/wake cycle | 7.9e-03 | Group18 | ALB, CRH, UTS2R |

Table S7. Significant GO terms in the 90 identified mammary gland/milk production-related genes.

| GO:ID | GO terms | *P*-value | Group | Associated genes |
| --- | --- | --- | --- | --- |
| GO:0030879 | mammary gland development | 4.21e-07 | Group0 | ATP2B2, B4GALT1, CSN3, FGF2, ID2, NME1, PTHLH, TFAP2C, TNFSF11, XDH |
| GO:0061180 | mammary gland epithelium development | 2.33e-03 | Group0 | FGF2, ID2, PTHLH, TFAP2C, TNFSF11 |
| GO:0007595 | lactation | 8.55e-03 | Group0 | ATP2B2, CSN3, NME1, XDH |
| GO:0007589 | body fluid secretion | 9.13e-03 | Group0 | ATP2B2, CSN3, NME1, XDH |
| GO:0032941 | secretion by tissue | 5.91e-03 | Group0 | ATP2B2, CSN3, NME1, XDH |
| GO:0002573 | myeloid leukocyte differentiation | 1.55e-03 | Group1 | CA2, CITED2, FAM20C, ID2, NME1, TNFSF11, UBD |
| GO:0061180 | mammary gland epithelium development | 2.33e-03 | Group1 | FGF2, ID2, PTHLH, TFAP2C, TNFSF11 |
| GO:0034103 | regulation of tissue remodeling | 1.48e-02 | Group1 | B4GALT1, CA2, TNFSF11, TP53 |
| GO:0033598 | mammary gland epithelial cell proliferation | 1.84e-02 | Group1 | ID2, TFAP2C, TNFSF11 |
| GO:0034105 | positive regulation of tissue remodeling | 2.06e-02 | Group1 | B4GALT1, CA2, TNFSF11 |
| GO:0002763 | positive regulation of myeloid leukocyte differentiation | 7.32e-03 | Group1 | CA2, ID2, TNFSF11 |
| GO:0002761 | regulation of myeloid leukocyte differentiation | 7.84e-03 | Group1 | CA2, ID2, NME1, TNFSF11 |
| GO:0045639 | positive regulation of myeloid cell differentiation | 3.41e-03 | Group1 | CA2, ID2, TNFSF11 |
| GO:0006633 | fatty acid biosynthetic process | 7.84e-04 | Group2 | ACSS1, ALOX12, ELOVL1, FASN, INSIG1, LPL, SCD |
| GO:0006641 | triglyceride metabolic process | 1.61e-03 | Group2 | ELOVL1, FASN, GK, INSIG1, LPL, TNXB |
| GO:0006639 | acylglycerol metabolic process | 2.15e-03 | Group2 | ELOVL1, FASN, GK, INSIG1, LPL, TNXB |
| GO:0006638 | neutral lipid metabolic process | 2.25e-03 | Group2 | ELOVL1, FASN, GK, INSIG1, LPL, TNXB |
| GO:0019432 | triglyceride biosynthetic process | 1.21e-02 | Group2 | ELOVL1, FASN, GK, LPL |
| GO:0046460 | neutral lipid biosynthetic process | 1.38e-02 | Group2 | ELOVL1, FASN, GK, LPL |
| GO:0046463 | acylglycerol biosynthetic process | 1.38e-02 | Group2 | ELOVL1, FASN, GK, LPL |
| GO:0030879 | mammary gland development | 4.21e-07 | Group4 | ATP2B2, B4GALT1, CSN3, FGF2, ID2, NME1, PTHLH, TFAP2C, TNFSF11, XDH |
| GO:0061180 | mammary gland epithelium development | 2.33e-03 | Group4 | FGF2, ID2, PTHLH, TFAP2C, TNFSF11 |
| GO:0033598 | mammary gland epithelial cell proliferation | 1.84e-02 | Group4 | ID2, TFAP2C, TNFSF11 |
| GO:0002763 | positive regulation of myeloid leukocyte differentiation | 7.32e-02 | Group4 | CA2, ID2, TNFSF11 |
| GO:0060443 | mammary gland morphogenesis | 1.56e-03 | Group4 | B4GALT1, PTHLH, TFAP2C |
| GO:0045639 | positive regulation of myeloid cell differentiation | 3.41e-03 | Group4 | CA2, ID2, TNFSF11 |
| GO:0032874 | positive regulation of stress-activated MAPK cascade | 2.93e-03 | Group5 | TNFSF11, TPD52L1, XDH |
| GO:0043506 | regulation of JUN kinase activity | 3.25e-02 | Group6 | DAB2, DVL2, TNFSF11, TNXB |
| GO:0060071 | Wnt signaling pathway, planar cell polarity pathway | 3.36e-02 | Group6 | DAB2, DVL2, RORA |
| GO:0090175 | regulation of establishment of planar polarity | 3.36e-02 | Group6 | DAB2, DVL2, RORA |
| GO:0035567 | non-canonical Wnt signaling pathway | 6.83e-03 | Group6 | DAB2, DVL2, RORA |
| GO:0043507 | positive regulation of JUN kinase activity | 1.90e-03 | Group6 | DAB2, DVL2, TNFSF11 |
| GO:2000425 | regulation of apoptotic cell clearance | 6.86e-05 | Group7 | CCL2, ITGAV, MFGE8 |
| GO:0043277 | apoptotic cell clearance | 1.65e-02 | Group7 | CCL2, ITGAV, MFGE8 |
| GO:0050766 | positive regulation of phagocytosis | 2.79e-02 | Group7 | CCL2, ITGAV, MFGE8 |
| GO:0045807 | positive regulation of endocytosis | 4.14e-02 | Group7 | CCL2, DAB2, ITGAV, MFGE8 |
| GO:0031058 | positive regulation of histone modification | 4.49e-03 | Group8 | CTR9, MUC1, PPARGC1A, TP53 |
| GO:2001252 | positive regulation of chromosome organization | 9.20e-03 | Group8 | CTR9, MUC1, PPARGC1A, TP53 |

Table S10. 40 genes in the intersection of XP-CLR, XP-EHH and conditional mutual information for Angus.

| Ensemble Gene | Gene Symbol | Description | Location |
| --- | --- | --- | --- |
| ENSBTAG00000002863 | ACAA2 | acetyl-CoA acyltransferase 2 | 24:49442439-49478083 |
| ENSBTAG00000001010 | ADAMTS18 | ADAM metallopeptidase with thrombospondin type 1 motif 18 | 18:4522898-4666475 |
| ENSBTAG00000001243 | AP3M2 | adaptor related protein complex 3 subunit mu 2 | 27:37031655-37049363 |
| ENSBTAG00000004844 | ARL2BP | ADP ribosylation factor like GTPase 2 binding protein | 18:25251821-25257892 |
| ENSBTAG00000022588 | C14H8orf34 | chromosome 14 C8orf34 homolog | 14:32378189-32741893 |
| ENSBTAG00000002907 | C2CD3 | C2 domain containing 3 centriole elongation regulator | 15:53454345-53526389 |
| ENSBTAG00000054229 | C2CD3 | C2 domain containing 3 centriole elongation regulator | 15:53552619-53579820 |
| ENSBTAG00000003189 | CCDC6 | coiled-coil domain containing 6 | 28:15463074-15571693 |
| ENSBTAG00000054245 | CD163L1 | CD163 molecule-like 1 | 5:103022590-103086215 |
| ENSBTAG00000045699 | CTNNA3 | catenin alpha 3 | 28:22282909-24121400 |
| ENSBTAG00000009187 | DNAJC28 | DnaJ heat shock protein family (Hsp40) member C28 | 1:2028900-2030042 |
| ENSBTAG00000006134 | DYNLRB1 | dynein light chain roadblock-type 1 | 13:63888186-63905671 |
| ENSBTAG00000003687 | FOXK2 | forkhead box K2 | 19:50030249-50079424 |
| ENSBTAG00000012501 | FTO | FTO alpha-ketoglutarate dependent dioxygenase | 18:22040310-22464466 |
| ENSBTAG00000010505 | INTS4 | integrator complex subunit 4 | 29:18041194-18149447 |
| ENSBTAG00000043990 | KHDRBS2 | KH RNA binding domain containing, signal transduction associated 2 | 23:270838-961226 |
| ENSBTAG00000006135 | MAP1LC3A | microtubule associated protein 1 light chain 3 alpha | 13:63918996-63920656 |
| ENSBTAG00000014855 | MICAL2 | microtubule associated monooxygenase, calponin and LIM domain containing 2 | 15:40374952-40604561 |
| ENSBTAG00000005945 | NXPE4 | neurexophilin and PC-esterase domain family, member 4 | 15:25080841-25098492 |
| ENSBTAG00000006313 | OR6C76 | olfactory receptor, family 6, subfamily C, member 76 | 5:58516084-58517061 |
| ENSBTAG00000009194 | OSBPL10 | oxysterol binding protein like 10 | 22:6157369-6498868 |
| ENSBTAG00000000837 | PARP1 | poly(ADP-ribose) polymerase 1 | 16:29365008-29406357 |
| ENSBTAG00000000700 | PARVA | parvin alpha | 15:40167676-40347859 |
| ENSBTAG00000024542 | PIGC | phosphatidylinositol glycan anchor biosynthesis class C | 16:39920499-39922955 |
| ENSBTAG00000011620 | PIGU | phosphatidylinositol glycan anchor biosynthesis class U | 13:63920854-64011921 |
| ENSBTAG00000001244 | PLAT | plasminogen activator, tissue type | 27:37053715-37077724 |
| ENSBTAG00000011700 | PLLP | plasmolipin | 18:25260164-25290238 |
| ENSBTAG00000027612 | PPME1 | protein phosphatase methylesterase 1 | 15:53580198-53629168 |
| ENSBTAG00000004718 | PUS7L | pseudouridine synthase 7 like | 5:36712229-36730075 |
| ENSBTAG00000015793 | RAB38 | RAB38, member RAS oncogene family | 29:7501290-7566916 |
| ENSBTAG00000020268 | RSF1 | remodeling and spacing factor 1 | 29:18188931-18332349 |
| ENSBTAG00000004842 | RSPRY1 | ring finger and SPRY domain containing 1 | 18:25212511-25249920 |
| ENSBTAG00000045243 | SCARNA18 | small Cajal body-specific RNA 18 | 7:82924661-82924798 |
| ENSBTAG00000017775 | SDCCAG8 | serologically defined colon cancer antigen 8 | 16:33638871-33884332 |
| ENSBTAG00000019041 | TAFA1 | TAFA chemokine like family member 1 | 22:32966389-33471943 |
| ENSBTAG00000032657 | TEAD1 | TEA domain transcription factor 1 | 15:39748793-40015294 |
| ENSBTAG00000046256 | TMEM132C | transmembrane protein 132C | 17:48417339-48868560 |
| ENSBTAG00000001258 | TMEM50B | transmembrane protein 50B | 1:2046733-2083566 |
| ENSBTAG00000033290 | TMEM60 | transmembrane protein 60 | 4:43450254-43454105 |
| ENSBTAG00000008414 | ZFR | zinc finger RNA binding protein | 20:41213852-41297142 |

Table S11. 55 genes in the intersection of XP-CLR, XP-EHH and conditional mutual information for Jersey

| Ensemble Gene | Gene Symbol | Description | Location |
| --- | --- | --- | --- |
| ENSBTAG00000006326 | ALDH1L2 | aldehyde dehydrogenase 1 family member L2 | 5:68542281-68603070 |
| ENSBTAG00000003111 | ATM | ATM serine/threonine kinase | 15:17853846-17996481 |
| ENSBTAG00000033547 | C17H4orf45 | chromosome 17 C4orf45 homolog | 17:39983282-40116757 |
| ENSBTAG00000049002 | C1D | C1D nuclear receptor corepressor | 11:66493584-66514419 |
| ENSBTAG00000013057 | CBLB | Cbl proto-oncogene B | 1:50258002-50481552 |
| ENSBTAG00000027875 | CCDC141 | coiled-coil domain containing 141 | 2:17799007-18020703 |
| ENSBTAG00000037844 | CDH18 | cadherin 18 | 20:53333437-53936986 |
| ENSBTAG00000009470 | CLIC4 | chloride intracellular channel 4 | 2:128114762-128188876 |
| ENSBTAG00000013662 | COL8A1 | collagen type VIII alpha 1 chain | 1:43902264-44073696 |
| ENSBTAG00000002765 | CYP24A1 | cytochrome P450, family 24, subfamily A, polypeptide 1 | 13:81592182-81610449 |
| ENSBTAG00000024605 | DYNLL1 | dynein light chain LC8-type 1 | 17:62791053-62793922 |
| ENSBTAG00000004287 | EVC | EvC ciliary complex subunit 1 | 6:103394278-103497766 |
| ENSBTAG00000004277 | EVC2 | EvC ciliary complex subunit 2 | 6:103500611-103662790 |
| ENSBTAG00000043989 | EYA3 | EYA transcriptional coactivator and phosphatase 3 | 2:125249517-125390286 |
| ENSBTAG00000025220 | FAM155A | family with sequence similarity 155 member A | 12:82805244-83413896 |
| ENSBTAG00000000820 | GNG11 | G protein subunit gamma 11 | 4:11242533-11247552 |
| ENSBTAG00000002674 | GNGT1 | G protein subunit gamma transducin 1 | 4:11210209-11235219 |
| ENSBTAG00000017086 | GRB10 | growth factor receptor bound protein 10 | 4:5106065-5327013 |
| ENSBTAG00000015683 | HSPA4 | heat shock protein family A (Hsp70) member 4 | 7:44607161-44661126 |
| ENSBTAG00000015727 | IFI47 | interferon gamma inducible protein 47 | 7:40459455-40475731 |
| ENSBTAG00000031654 | KCND3 | potassium voltage-gated channel subfamily D member 3 | 3:31311513-31530748 |
| ENSBTAG00000005477 | LAPTM5 | lysosomal protein transmembrane 5 | 2:122807359-122833480 |
| ENSBTAG00000003479 | MATN1 | matrilin 1 | 2:122848955-122858265 |
| ENSBTAG00000005888 | MDGA1 | MAM domain containing glycosylphosphatidylinositol anchor 1 | 23:11546127-11611239 |
| ENSBTAG00000037784 | MGC157405 | pregnancy-associated glycoprotein | 29:39124148-39133288 |
| ENSBTAG00000018155 | MRPS18C | mitochondrial ribosomal protein S18C | 6:98226288-98231259 |
| ENSBTAG00000022991 | NBEA | neurobeachin | 12:25769802-26441648 |
| ENSBTAG00000000220 | NEK10 | NIMA related kinase 10 | 22:1485764-1600233 |
| ENSBTAG00000013048 | NIPAL3 | NIPA like domain containing 3 | 2:128445501-128496459 |
| ENSBTAG00000020159 | NKAIN1 | sodium/potassium transporting ATPase interacting 1 | 2:122393085-122438183 |
| ENSBTAG00000045644 | OR2T6 | olfactory receptor, family 2, subfamily T, member 6 | 7:42054420-42055379 |
| ENSBTAG00000002138 | PADI1 | peptidyl arginine deiminase 1 | 2:135349139-135393383 |
| ENSBTAG00000012043 | PADI3 | peptidyl arginine deiminase 3 | 2:135316332-135346269 |
| ENSBTAG00000019173 | PAG12 | pregnancy-associated glycoprotein 12 | 29:37655999-37666176 |
| ENSBTAG00000047141 | PAG7 | pregnancy-associated glycoprotein 7 | 29:37942844-38048511 |
| ENSBTAG00000013801 | PBX1 | PBX homeobox 1 | 3:4147294-4246502 |
| ENSBTAG00000016026 | PCOLCE2 | procollagen C-endopeptidase enhancer 2 | 1:126132067-126231903 |
| ENSBTAG00000008812 | PFDN4 | prefoldin subunit 4 | 13:81632576-81644895 |
| ENSBTAG00000007635 | PLCL1 | phospholipase C like 1 (inactive) | 2:86319697-86686609 |
| ENSBTAG00000010604 | PNO1 | partner of NOB1 homolog | 11:66589551-66598955 |
| ENSBTAG00000010619 | PPP3R1 | protein phosphatase 3 regulatory subunit B, alpha | 11:66604007-66667996 |
| ENSBTAG00000007937 | PRIM2 | DNA primase subunit 2 | 23:2560548-2894440 |
| ENSBTAG00000014947 | PTPN13 | protein tyrosine phosphatase non-receptor type 13 | 6:101531746-101735421 |
| ENSBTAG00000018979 | PUM1 | pumilio RNA binding family member 1 | 2:122527753-122653986 |
| ENSBTAG00000017452 | RCAN3 | RCAN family member 3 | 2:128388785-128422500 |
| ENSBTAG00000050474 | RUNX3 | RUNX family transcription factor 3 | 2:127998304-128061752 |
| ENSBTAG00000008766 | SLC9A1 | solute carrier family 9 member A1 | 2:126090279-126142300 |
| ENSBTAG00000004094 | SPARCL1 | SPARC like 1 | 6:102370630-102423199 |
| ENSBTAG00000013281 | SPATA21 | spermatogenesis associated 21 | 2:135698556-135736414 |
| ENSBTAG00000010278 | STPG1 | sperm tail PG-rich repeat containing 1 | 2:128500788-128561411 |
| ENSBTAG00000001651 | SYF2 | SYF2 pre-mRNA splicing factor | 2:127725940-127731258 |
| ENSBTAG00000001296 | TMEM50A | transmembrane protein 50A | 2:127661915-127677708 |
| ENSBTAG00000018415 | TTLL4 | tubulin tyrosine ligase like 4 | 2:106656154-106706020 |
| ENSBTAG00000010595 | WDR92 | WD repeat domain 92 | 11:66560111-66589114 |
| ENSBTAG00000030616 | ZCCHC10 | zinc finger CCHC-type containing 10 | 7:44567086-44580862 |


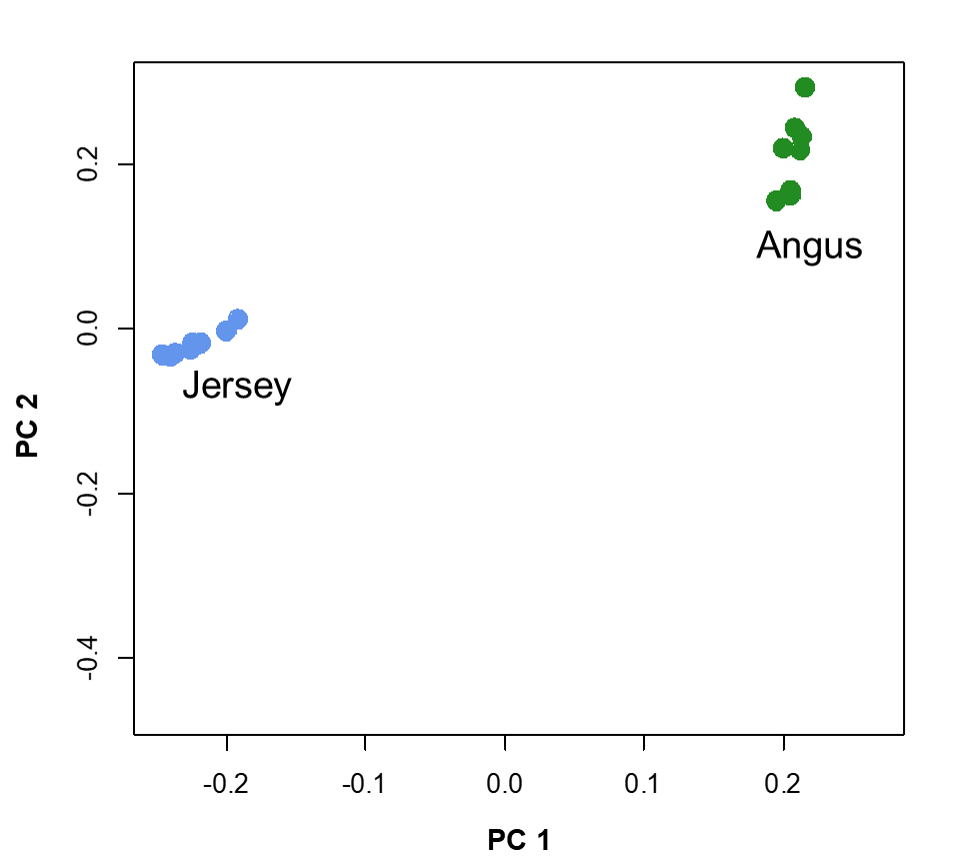


**Figure S1. Projection of 20 Angus and Jersey individuals plotted on the top two principal components**

The proportions of variance that explained the top two principal components were 19.06% and 8.24%. The samples are color coded to indicate each breed.


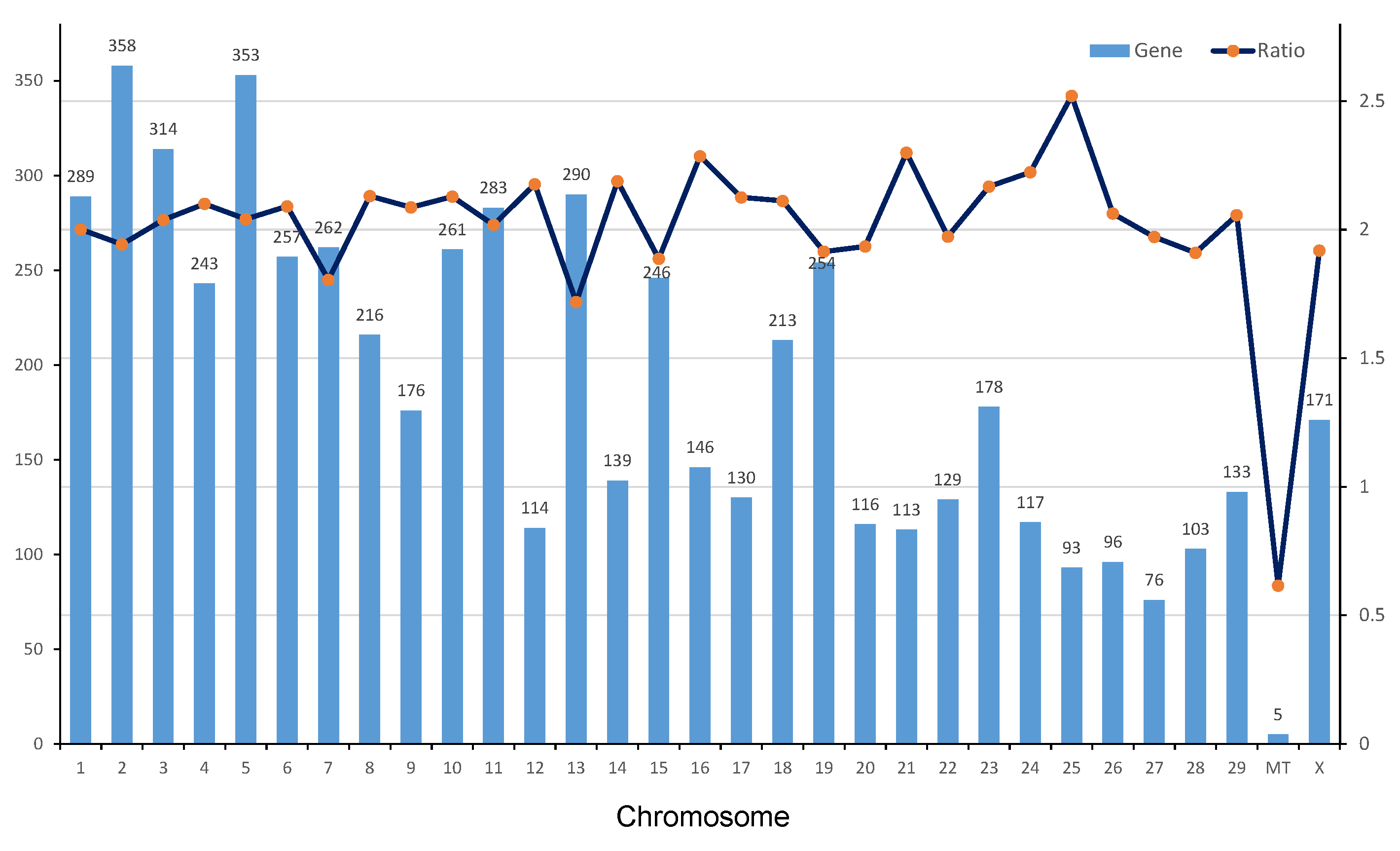


**Figure S2.** **Ratio of the identified SNPs to all the SNPs on each chromosome and the number of genes including the identified SNPs**

The ratio is negatively log-scaled; thus, a lower value indicates a higher ratio value.


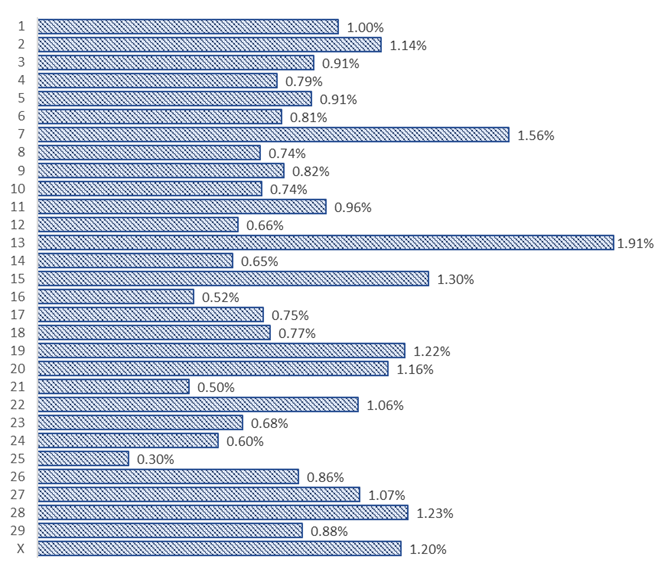


**Figure S3. Distributions of the ratio of the identified SNPs on each chromosome**

The distributions of the ratio (%) of the SNPs identified by CMI with a significant *p*-value (1.0$\times$10^-3^) to total the SNPs across all chromosomes excluding mitochondrial genome.

**# Heterozygosity**

**SNP position**

**Figure S4.** **Distribution of 858 SNPs for heterozygosity in the identified lipid/intramuscular fat-related genes in Angus versus Jersey cattle**

**SNP position**

**# Heterozygosity**

**Figure S5.** **Distribution of 852 SNPs for heterozygosity in the identified milk production-related genes in Angus versus Jersey cattle**


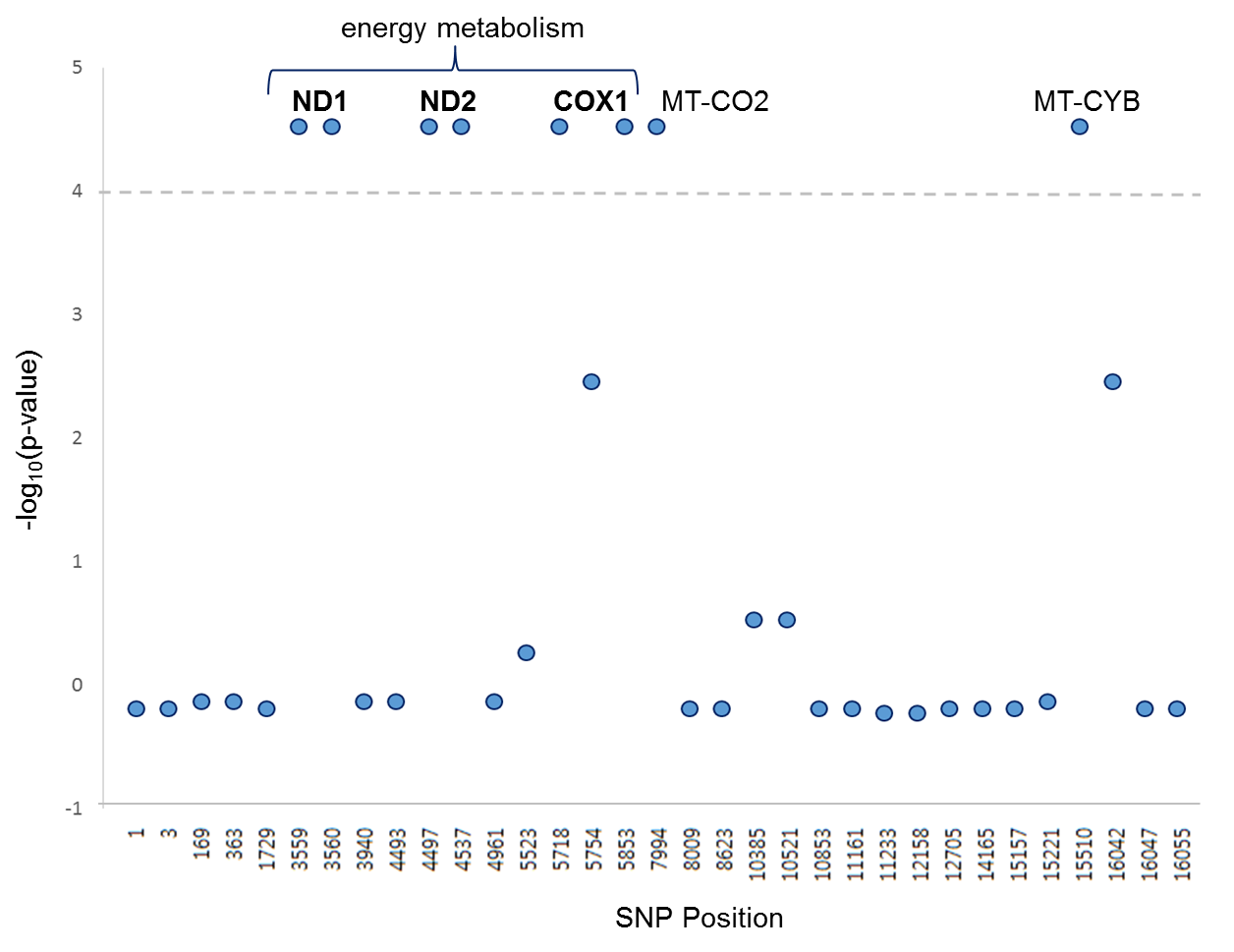


**Figure S6**. **33 SNPs on the mitochondrial genome plotted with negative log-scaled *p*-values**

Five genes were identified with eight SNPs that exceeded a significant threshold of 1.0$\times$10^-4^.

**
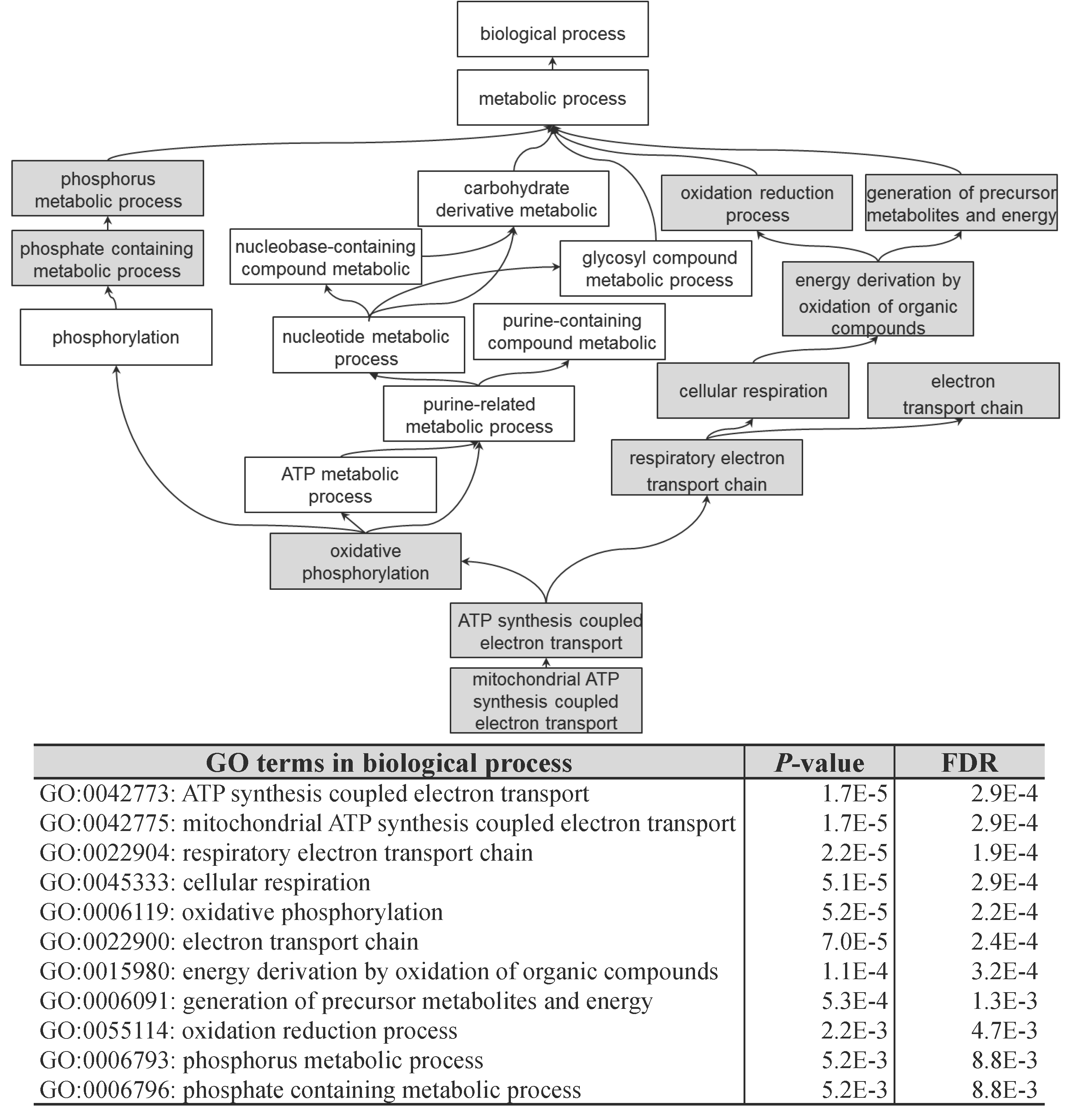
**

**Figure S7. Significant GO terms for ND1, ND2 and COX1 on the mitochondrial genome**

The top figure represents a subgraph of the GO to show the relationships among the enriched GO terms of the three genes, and the gray box denotes the overrepresented GO terms at significant levels (false discovery rate, FDR < 0.01).


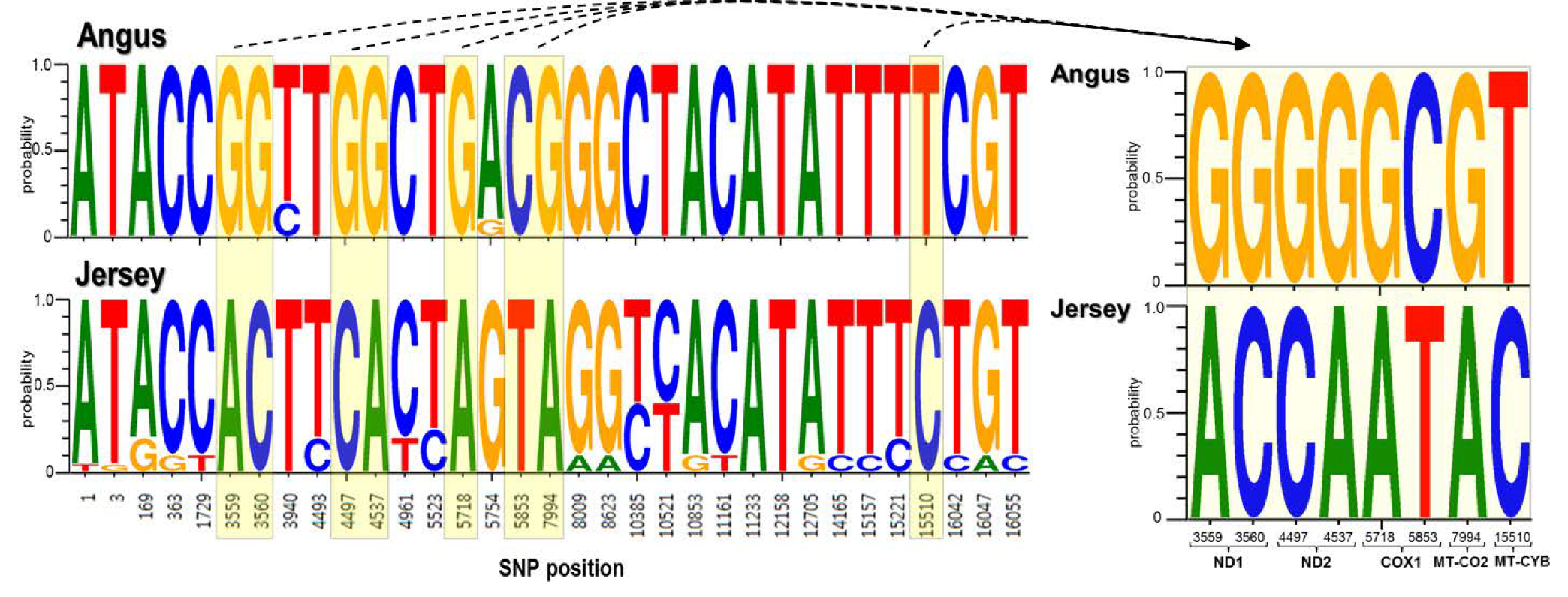


**Figure S8.** **Genotype profiles for each SNP locus on the mitochondrial genome** **of Angus and Jersey breeds**

The highlighted areas indicate the identified SNPs on the mitochondrial genome. The figure on the right represents the concatenation of the highlighted SNP areas.
